# Clinical and biomarker analyses of sintilimab plus gemcitabine and cisplatin as first-line treatment for patients with advanced biliary tract cancer

**DOI:** 10.1038/s41467-023-37030-w

**Published:** 2023-03-11

**Authors:** Tian-mei Zeng, Guang Yang, Cheng Lou, Wei Wei, Chen-jie Tao, Xi-yun Chen, Qin Han, Zhuo Cheng, Pei-pei Shang, Yu-long Dong, He-ming Xu, Lie-ping Guo, Dong-sheng Chen, Yun-jie Song, Chuang Qi, Wang-long Deng, Zhen-gang Yuan

**Affiliations:** 1 Department of Oncology, Eastern Hepatobiliary Surgery Hospital, Second military medical univercity, Shanghai, China; 2 Jiangsu Simcere Diagnostics Co., Ltd, The State Key Laboratory of Translational Medicine and Innovative Drug Development, Nanjing, China

**Keywords:** Biliary tract cancer, Predictive markers, Predictive markers

## Abstract

The prognosis of biliary tract cancer (BTC) remains unsatisfactory. This single-arm, phase II clinical trial (ChiCTR2000036652) investigated the efficacy, safety, and predictive biomarkers of sintilimab plus gemcitabine and cisplatin as the first-line treatment for patients with advanced BTCs. The primary endpoint was overall survival (OS). Secondary endpoints included toxicities, progression-free survival (PFS), and objective response rate (ORR); multi-omics biomarkers were assessed as exploratory objective. Thirty patients were enrolled and received treatment, the median OS and PFS were 15.9 months and 5.1 months, the ORR was 36.7%. The most common grade 3 or 4 treatment-related adverse events were thrombocytopenia (33.3%), with no reported deaths nor unexpected safety events. Predefined biomarker analysis indicated that patients with homologous recombination repair pathway gene alterations or loss-of-function mutations in chromatin remodeling genes presented better tumor response and survival outcomes. Furthermore, transcriptome analysis revealed a markedly longer PFS and tumor response were associated with higher expression of a 3-gene effector T cell signature or an 18-gene inflamed T cell signature. Sintilimab plus gemcitabine and cisplatin meets pre-specified endpoints and displays acceptable safety profile, multiomics potential predictive biomarkers are identified and warrant further verification.

## Abstract (web summary)

Chemotherapeutic agents and immune checkpoint inhibitors have shown modest efficacy in patients with advanced biliary tract cancers (BTCs). Here the authors report the results of a phase II trial of the anti-PD1 antibody sintilimab plus chemotherapy (gemcitabine and cisplatin) as first-line treatment for patients with advanced BTCs.

## Introduction

Biliary tract cancer (BTC) is a heterogeneous hepatobiliary malignant tumor, comprising cholangiocarcinoma (tumors originating from the intrahepatic, perihilar, or distal biliary tree) and gallbladder carcinoma^1^. The incidence of BTC varies between subgroups and geographical regions^1^. Additionally, the incidence of BTC has been rising worldwide in recent years^2^. Surgery remains the only curative treatment option for BTC, however, it is not amenable for most patients (70%) with unresectable or metastatic disease as they are asymptomatic in the early stages. In such cases, the 5-year survival rate is less than 5%, and the recurrence rate remains high even after radical surgery^3,4^.

The ABC-02 study is the landmark study to establish gemcitabine and cisplatin (GemCis) as the standard first-line therapy for BTC, based on its superior overall survival benefits [11.7 vs. 8.1 months; hazard ratio (HR): 0.64, 95% confidence interval (CI): 0.52–0.80, *p* < 0.001] compared with gemcitabine alone^5^. Many other novel chemotherapy agents have presented modest efficacy, but the overall benefits are still limited^6^. Hence, there is an urgent requirement to develop other treatment options.

Immune checkpoint inhibitors (ICIs) have shown clinical benefits in various cancers, such as lung cancer, malignant melanoma, and renal carcinoma. The antitumor activity of anti-programmed cell death 1/ligand 1 (PD-1/PD-L1) antibodies monotherapy in BTC has been investigated, including pembrolizumab and nivolumab in patients with BTC. However, the efficacy is relatively modest (objective response rates (ORRs) range from 3–22%)^7,8^. Chemotherapeutic agents have been shown to induce immunomodulatory effects^9,10^. Durvalumab plus GemCis has depicted significantly better overall survival (OS) benefits compared with GemCis alone (12.8 vs. 11.5 months; HR: 0.80, 95% CI: 0.66–0.97, *p* = 0.021) in TOPAZ-1 trial^11^. Based on these OS results, durvalumab plus GemCis is recommended as one of the first-line regimens for unresectable and metastatic BTC by the national comprehensive cancer network guidelines 2022. However, the ICIs used in this study target PD-L1, which delivers different efficacy and safety profile from anti-PD-1^12^. Therefore, the combination of PD-1inhibitor plus chemotherapy still warrants further exploration.

Currently, favorable biomarkers are lacking for the immunotherapy of BTC. There is no definitive evidence that positive PD-L1 expression^13–15^ or tumor mutation burden (TMB) contribute to predicting which patients might benefit most from the use of ICIs^16^. Although efforts have been made to reveal specific genetic and immunologic characteristics to determine the prognostic value of ICIs in BTCs, especially in those who received immune-combined therapy, the progress is slow.

In this work, we conduct this prospective trial to evaluate the efficacy and safety of sintilimab, a selective anti-PD-1 antibody approved by the national medical products administration of China for treating Hodgkin lymphoma and hepatocellular carcinoma, in combination with GemCis for advanced BTCs. We also investigate the potential predictive biomarkers of clinical response.

## Results

### Workflow of the study

**Fig. 1:**
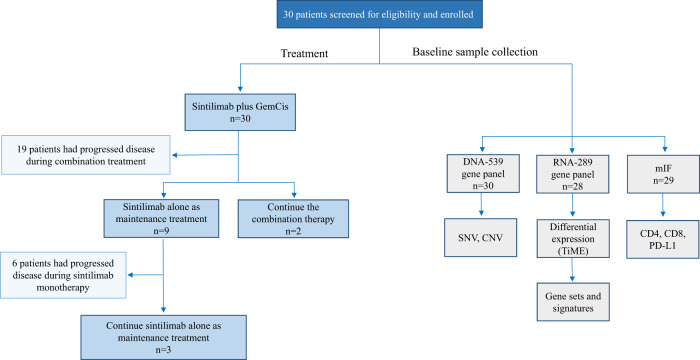
Clinical trial flow chart. Flow of participants in the study.

Between August 2020 and May 2022, 30 patients were enrolled, which constituted the intention-to-treat population. Until the data cutoff (May 08, 2022; end of the follow-up period), the median follow-up duration was 12.3 months (95% CI: 9.1–16.0), and the median treatment cycle for sintilimab plus chemotherapy was 5.5 (range: 1–8). Fifteen (50%) patients received 6–8 cycles of combined therapy, whereas the remaining 15 patients received less than 6 cycles of treatment. The median treatment duration was 4.3 months (range: 0.7–7.1) for chemotherapy and 4.9 months (range: 0.7–20.1) for sintilimab. Eighteen patients experienced disease progression during the combination period, while nine completed 6–8 cycles of treatment in the combination regimens and used sintilimab alone as maintenance therapy; during this period, six patients experienced disease progression and three were still undergoing sintilimab treatment. In total, 5 (17%) patients continued the study treatment without disease progression (Fig. 1).

### Patients’ characteristics

The baseline demographics of the enrolled patients are presented in Table 1. The median age was 56.5 years (range: 35–73). Patients with an Eastern Cooperative Oncology Group performance score of 0–1 accounted for 96.66% (29/30) of patients; 18 (60%) of the 30 patients were male, while 25 (83.3%) had metastatic disease. Intrahepatic cholangiocarcinoma was mostly found to be of the primary tumor type (28/30, 93.3%). All patients presented microsatellite Supplementary Table (MSS) by immunohistochemistry (IHC) staining or NGS-based panel testing, part of the IHC staining results were presented in Supplementary Fig 1.

**Table 1:** Patient demographics and baseline characteristics

Characteristics	*n* = 30
Median age, years	56.5 (35–73)
Gender, *n*(%)	
male	18 (60.00%)
female	12 (40.00%)
Eastern Cooperative Oncology Group performance status, *n* (%)	
0	2 (6.67%)
1	27 (90.00%)
2	1 (3.33%)
Cholangiocarcinoma location, *n*(%)	
Intrahepatic cholangiocarcinoma	28 (93.33%)
Extrahepatic cholangiocarcinoma	2 (6.67%)
Pathological type, *n*(%)	
Adenocarcinoma	27 (90.00%)
Squamous cell carcinoma	1 (3.33%)
Others	2 (6.67%)
Clinical stage, *n*(%)	
II	2 (6.67%)
IIIB	4 (13.33%)
IVB	24 (80.00%)
Metastasis, *n*(%)	
Yes	25 (83.33%)
Metastatic site, *n*(%)	
Lymphonodus	17 (56.67%)
Bone	7 (23.33%)
Peritoneum	4 (13.33%)
Liver	3 (10.00%)
Lung	3 (10.00%)
Adrenal gland	1 (3.33%)
pelvic cavity	1(3.33%)
Microsatellite stability status, *n*(%)	
microsatellite stable	30 (100%)

### Efficacy

**Fig. 2:**
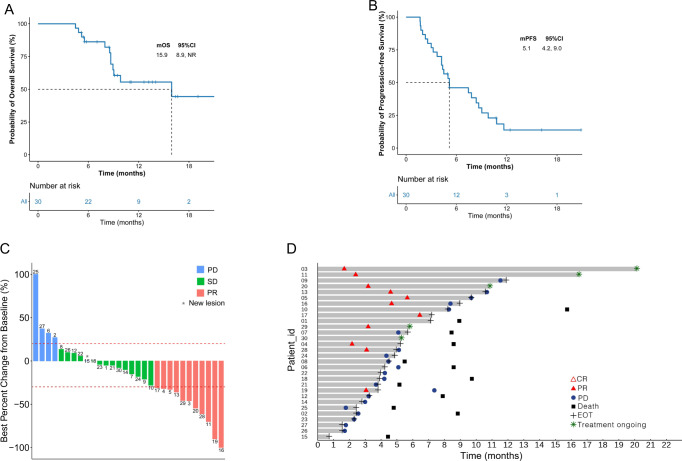
Kaplan-Meier curves and characteristics of objective response in the study. **A** Kaplan-Meier estimates of overall survival (*n* = 30). **B** Kaplan-Meier estimates of progression-free survival by RECISTv1.1 (*n* = 30). **C** Change of target lesion in tumor size from baseline to best response (*n* = 30). **D** Swimming chart showing the treatment results (*n* = 30), the length of each bar represents the duration of treatment of each patient in the IIT population. Source data are provided as a Source Data file. PR partial response, SD Supplementary Table disease, PD progressive disease.

By the end of the study period, the median OS was 15.9 months (95% CI: 8.9–not reached, Fig. 2A). Data were immature and censored for 18 (60%) patients. The OS rate at 6 and 12 months was 85.6% (95% CI: 65.9–94.4) and 54.5% (95% CI: 32.6–72.1), respectively. The median PFS was 5.1 months (95% CI: 4.2–9.0, Fig. 2B), and the 6- and 12-month PFS were 45.3% (95% CI: 26.8–62.1) and 13.6% (95% CI: 3.7–29.8), respectively. Among the patients who had completed at least one tumor response evaluation, 11 (36.7%) achieved PR, 14 (46.7%) had SD, and 5 (16.7%) were disease progression (PD). In this study, the ORR was 36.7%, while the DCR was 83.3%. The best changes in comparison to the baseline tumor size are shown in Fig. 2C, and the overall treatment results are depicted using a swimmer chart in Fig. 2D. The median duration of response was 6.2 months (95% CI: 2.1–not reached) for patients who achieved an objective response.

### Safety

Safety data reported during the trial are summarized in Table 2. Treatment-related adverse events of any grade were reported in all patients. Here, thrombocytopenia was reported as the most common (10 patients; 33.3%) grade 3 or 4 treatment-related adverse event. No treatment-related deaths were reported. Sintilimab was discontinued due to a treatment-related adverse event in 1 (3.3%) patient, and 14 (46.7%) patients had a dose reduction. The most frequent immune-mediated adverse event was hypothyroidism (8 patients; 26.7%). Additionally, 6 of the 30 patients (20%) developed serious adverse events, but all of them were relieved by dose reduction and discontinuation.

**Table 2:** Adverse events in all treated patients

AE, *n*(%)	*n* = 30
Any TEAE	30 (100%)
Any TRAE	30 (100%)
3-4 grade TEAE	18 (60%)
3-4 grade TRAE	17 (56.7%)
SAE	6 (20.0%)
Treatment-related SAE	6 (20.0%)
Drug withdraw	
Chemotherapy-related drug withdraw	0 (0%)
Sintilimab-related drug withdraw	1 (3.3%)
Dose reduction caused by TRAE	14 (46.7%)
Treatment-related death	0 (0%)
Any grade irAE	12 (40,0%)
3-4 grade TRAE	
Thrombocytopenia	10 (33.3%)
Agranulocytosis	3 (10.0%)
leukopenia	3 (10.0%)
Neutropenia	2 (6.7%)
Hypothyroidism*	2 (6.7%)
Anemia	1 (3.3%)
Elevated transaminase	1 (3.3%)
Autoimmune encephalitis*	1 (3.3%)

*AE* adverse event, *TEAE* treatment-emergent adverse event, *TRAE* treatment-related adverse event, *SA*E serious adverse event, irAE immune-related adverse event.

*immune-related adverse event

### Overview of the genomic mutation spectrum

**Fig. 3:**
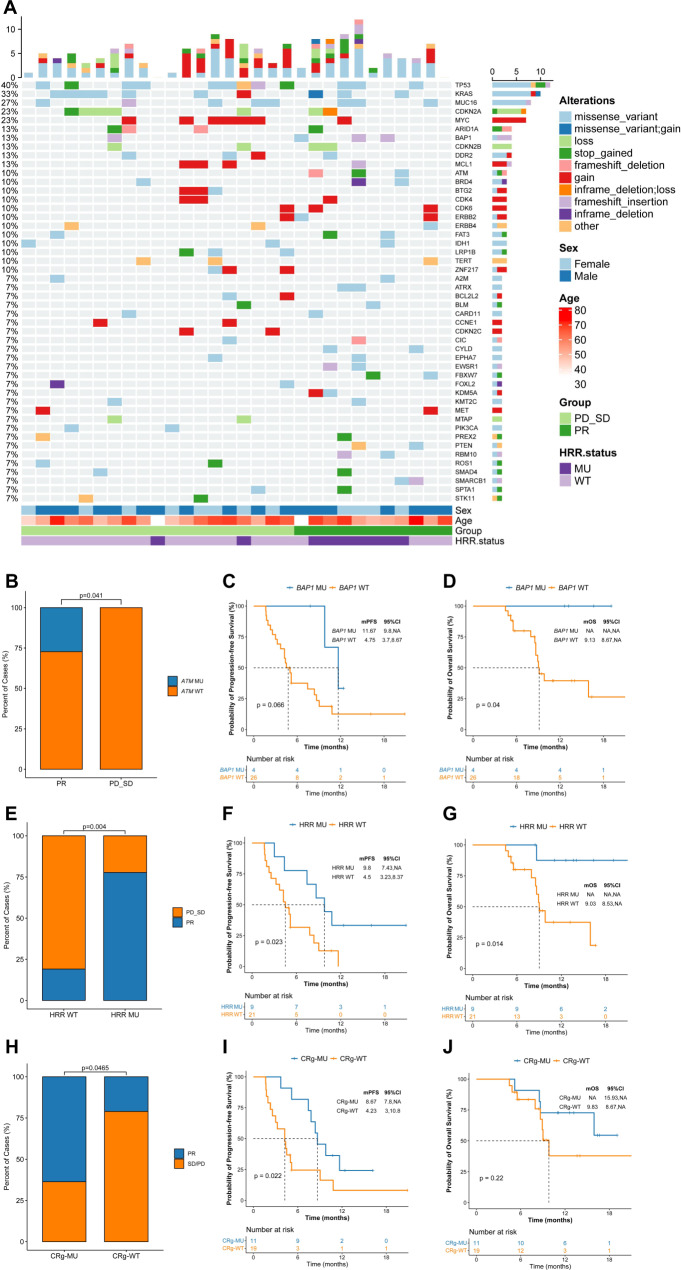
Overview of the genomic mutation spectrum and association between gene alterations and clinical response. **A** Overview of the genomic mutation spectrum in the cohort (*n* = 30). **B** Higher *ATM* mutation rate in the responding group, 3 patients with *ATM* mutation in the responding group (*n* = 11), and no patients with *ATM* mutation in the non-responding group (*n* = 19). Fisher’s exact test was used to determine statistical significance between the two groups. **C**, **D** *BAP1* mutation showed longer survival trend in the association with PFS and OS. *p*-values were based on a two-sided log-rank test. **E** HRR pathway gene alteration presented superior ORR, 7 patients achieved PR in the HRR pathway gene alteration group (*n* = 9), and 4 patients achieved PR in the HRR pathway gene wild-type group (*n* = 21). Fisher’s exact test was used to determine statistical significance between the two groups. **F**, **G** HRR pathway gene alteration presented superior survival outcome, *p*-values were based on a two-sided log-rank test. **H** loss-of-function mutations in chromatin remodeling genes presented superior ORR, 7 patients achieved PR in the chromatin remodeling genes alteration group (*n* = 11), and 4 patients achieved PR in the chromatin remodeling genes wild-type group (*n* = 19). Fisher’s exact test was used to determine statistical significance between the two groups. **I**, **J** loss-of-function mutations in chromatin remodeling genes presented superior survival outcome, *p*-values were based on a two-sided log-rank test. (CRg: chromatin remodeling genes). Source data are provided as a Source Data file.

In the prespecified exploratory analysis, we first evaluated the association between genomic alteration and clinical response to sintilimab combined with GemCis in all the enrolled participants with pretreatment tumor biopsies for targeted 539 cancer-relevant genes sequencing. Fig 3A depicts the genetic alterations in the entire cohort. *TP53* was the most frequently altered gene, occurring in 12 (40%) patients, including 8 cases with missense mutations. *KRAS* was altered in 10 (33%) patients, followed by *MUC16* (27%), *CDKN2A* (23%), and *MYC* (23%). The median TMB was 3.3 muts/mb in responders (PR) versus 2.3 muts/mb in non-responders (SD + PD) (*p* = 0.38, Supplementary Fig 2A). No association was observed for PFS (high vs. low: 5.17 vs. 5.0 months, *p* = 0.56; Supplementary Fig 2B) or OS (high vs. low: 15.9 vs. not reached, *p* = 0.99; Supplementary Fig 2C) between the TMB-high (median split, ≥3.55 mut/Mb) and -low groups (<3.55 mut/Mb).

### Association between gene alterations and clinical response

We next sought to assess whether the response to combined therapy was associated with mutations in specific genes or pathways. The distribution of single-nucleotide variants associated with tumor response and survival outcome is depicted in Supplementary Table 1. Notably, patients in the responding group had a significantly higher ataxia telangiectasia mutated (*ATM*) mutation rate (27.2% vs. 0%, *p* = 0.041; Fig. 3B). Additionally, *BRCA1*-associated protein (*BAP1*) mutation showed a longer survival trend in association with PFS (mutant vs. wild-type: 11.6 vs. 4.7 months, *p* = 0.066; Fig. 3C) and OS (mutant vs. wild-type: not reached vs. 9.1 months, *p* = 0.04; Fig. 3D). Considering that *ATM* and *BAP1* are homologous recombination repair (HRR)-related genes, we then investigated whether the response to therapy was associated with a gene mutation in the HRR pathway. Remarkably, HRR pathway gene alteration presented superior survival outcomes and ORR. The ORR was 77.8% versus 19% in HRR mutant and wild-type groups (*p* = 0.004; Fig. 3E), respectively. The PFS and OS were 9.8 versus 4.5 months (*p* = 0.023; Fig. 3F) and not reached versus 9 months (*p* = 0.014; Fig. 3G), respectively. Interestingly, 2 of 4 patients with *BAP1* mutation also harbored *ATM* mutation. However, whether these synergistic co-mutations account for the substantial clinical benefit in patients with *BAP1* mutations cannot be determined in our limited sample study. Moreover, germline or somatic *BRCA1/2*, *PALB2* alterations, somatic *BLM* and *RECQL4* mutations and some other HRR pathway genes were also analyzed in our study. However, due to the low proportion of mutations in these genes, there was no significant difference in the proportion of mutations in these genes between the responding and non-responding groups, and there was no significant correlation between these genes mutation and survival prognosis (Supplementary Table 1). Given that genetic mutation detection varies with the sequencing depth and the quality of the sample, transcriptomic signatures have better predictive power than mutational signatures in some settings^17^. Thus, the association between transcriptomic features of HRD and clinical outcomes were investigated, we found that the group with high HRD transcriptomic signature score tended to have better PFS compared to the low group (PFS: high vs. low: 7.8 vs. 4.3, HR: 0.57, *p* = 0.17; Supplementary Fig 2D), and no significant OS benefit was found in high HRD transcriptomic signature score compared to the low group (Supplementary Fig 2E).

As an recent study demonstrated that loss-of-function mutations in chromatin remodeling genes may be associated with longer survival in patients with BTCs receiving chemo-immunotherapy^18^, we also evaluated this potential predictive biomarker in our study. We found that 36.7% (11/30) of patients harbored at least one inactivating mutation in genes of chromatin remodeling complex. The ORR was 63.6% versus 21.1% in chromatin remodeling genes mutant and wild-type groups (p = 0.046; Fig. 3H), patients with mutations in chromatin remodeling genes demonstrated a significantly longer PFS (8.7 months vs. 4.2 months; HR: 0.37; *p* = 0.021, Fig. 3I) and a trend for longer OS (not reached vs.9.8 months; HR: 0.47; *p* = 0.21, Fig. 3J). Regarding copy number variants, no association was observed between the total number of copy number variants (4.0 vs. 2.9, *p* = 0.96, Supplementary Fig 2F) in responders and non-responders. However, *CDKN2B* mutations showed a significant association with prolonged survival outcomes (PFS: mutant vs. wild-type: 11.6 vs. 5.0 months, *p* = 0.041, Supplementary Fig 2G; OS: mutant vs. wild-type: not reached vs. 9.8 months, *p* = 0.061, Supplementary Fig 2H).

### Identification of differentially expressed genes in responders and non-responders

**Fig. 4:**
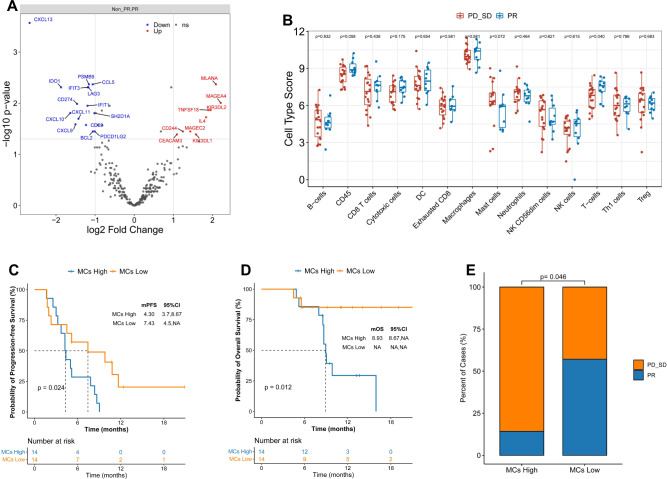
Identification of differentially expressed genes and immune cell profile analyses in responders and non-responders. **A** Transcriptome analysis on differential expression between responders (*n* = 10) and non-responders (*n* = 18), DESeq2 was provided to perform differential expression testing using the Wald test, multiple testing was adjusted using Benjamini and Hochberg method. **B** The abundance of predefined 14 immune cells composition between responders (*n* = 10) and non-responders (*n* = 18), box plots are indicated in terms of minima, maxima, centre, bounds of box and whiskers (interquartile range value), and percentile in the style of Tukey. Wilcoxon test was used to determine the statistical significance between subgroups. The adverse predictive value of tumor-infiltrating MCs in immuno-chemotherapy, *p*-values were based on a two-sided log-rank test for survival analysis. **E** Higher tumor-infiltrating MCs in immuno-chemotherapy presented worse ORR, 2 patients achieved PR in the high (median) tumor-infiltrating MCs group (*n* = 14), and 8 patients achieved PR in the low (median) tumor-infiltrating MCs group (*n* = 14). Fisher’s exact test was used to determine the statistical significance between the two groups. Source data are provided as a Source Data file.

To further assess the impact of the tumor immune microenvironment (TiME) on clinical response to sintilimab combined with GemCis, we performed extensive transcriptome analysis using NanoString nCounter assay in 28 of the 30 participants. The results of the transcriptome analysis on differential expression between responders and non-responders identified 24 significant genes (Fig. 4A). Here, 15 genes that are known to be associated with immunotherapy response, including those interferon-induced-related genes (*PSMB9*, *IFIT3*, and *IFIT1*); chemokines (*CXCL13*, *CCL5*, *CXCL10*, *CXCL11*, *CXCL9*), and immune checkpoint targets like CD274 (encodes PD-L1), IDO1, LAG3, and PDCD1LG2 (encodes PD-L2), were upregulated in responders. Conversely, expression levels of some carcinoembryonic antigen genes (*MLANA*, *MAGEA4*, *MAGEC2*, *CEACAM3*), MC-produced IL-4, and intratumoral myeloid-derived suppressor cell-associated *CD244* and killer cell immunoglobulin-like receptors (*KIR3DL1, KIR3DL2*) were upregulated in non-responders. Gene Ontology (GO) enrichment and Kyoto Encyclopedia of Genes and Genomes (KEGG) pathways analysis were performed to identify the molecular function of differentially expressed genes. T-cell activation, chemokine activity, chemokine receptor binding, and other related pathways were significantly enriched in GO analysis (Supplementary Fig 3A). Similar findings in KEGG analysis revealed that differential expression gene enrichment was concentrated in cytokine–cytokine receptor interaction and chemokine signaling pathways (Supplementary Fig 3B). Furthermore, we assessed the prognostic value of all these candidate genes included in the panel using Kaplan–Meier analysis; gene expression levels associated with survival outcomes are summarized in Supplementary Table 2.

Considering that serological carcinoembryonic antigen (CEA) is generally elevated in BTC patients, and up-regulation of CEA genes were also identified in our transcriptome analysis, so we further investigate this relationship in our cohort. Firstly, we evaluated the possibility of CEA level as a predictive biomarker by comparing baseline CEA levels between the two groups, we found that baseline CEA levels in non-responders were significantly higher than those in responders (non-responders vs. responders: 31.23 ± 12.10 vs. 3.89 ± 0.96, *p* = 0.036, Supplementary Fig 4A). Meanwhile, we also investigated the association between baseline CEA level and clinical response. Consistently, only one patients (1/9, 11.1%) achieved an objective response in the abnormal CEA group (≥10 ng/ml); this proportion was lower than that in the normal (≤10 ng/ml) CEA group (47.6%, 10/21, *p* = 0.1, Supplementary Fig 4B). These findings highlighted the adverse predictive value of elevated CEA level in immuno-chemotherapy for BTCs.

### Immune cell profile analyses in responders and non-responders

Subsequently, we assessed the abundance of 14 predefined immune cells based on the nCounter immune profile panel. At the metagene level, the score of total T cells was significantly decreased in non-responders compared to responders (6.9 vs. 7.7, *p* = 0.04; Fig. 4B). However, its significant association with prognosis was not observed (median, PFS: high vs. low: 6.3 vs. 4.6 months, *p* = 0.5; OS: high vs. low: not reached vs. 15.9 months, *p* = 0.86; Supplementary Fig 4C, D). Meanwhile, we found that the score of MCs was increased in non-responders compared with responders (7.4 vs. 6.8, *p* = 0.072). Further survival analysis showed that high levels of tumor-infiltrating MCs were associated with markedly shorter survival outcomes in PFS (median, high vs. low: 4.3 vs. 7.4 months, *p* = 0.024) and OS (high vs. low: 8.9 months vs. not reached, *p* = 0.012; Fig. 4C, D). Only 14.2% (2/14) of the patients achieved an objective response in the high MCs score group; this proportion was significantly lower than that in the low MCs score group (57.1%, 8/14, *p* = 0.046; Fig. 4E). These findings highlighted the adverse predictive value of tumor-infiltrating MCs in immuno-chemotherapy for BTCs.

In a previous report, accumulation of progenitor-exhausted T cells was found as an important mechanism in response to anti-PD1/PD-L1 therapy^19^. Thus, the role of exhausted T cell in the clinical outcomes were investigated in our study. Considering exhausted T cells refer to a heterogenous group containing multiple stages, we focused on the status and clinical outcome correlation of baseline terminally exhausted T cells (Tex^term^ cells) and progenitor exhausted T cells (Tex^pro^ cells). With regard to Tex^term^ cells, we found that the patients with high Tex^term^ cells signature score tended to have better OS compared to the low group, but no significant PFS benefit was found in high Tex^term^ cells score compared to the low group (median split, PFS: high vs. low: 4.7 vs. 5.0, *p* = 0.35; OS: high vs. low: Not reached vs. 9.0, HR: 0.61, *p* = 0.4; Supplementary Fig 4, E, F). Similarly, patients with Tex^term^ cells signature score-high had a higher objective response rate (ORR: high vs. low: 50% vs. 20%, *p* = 0.23; Supplementary Fig 4G), but with no statistically significant difference. In term of Tex^pro^ cells, we found patients with low Tex^pro^ cells signature score seems to be a better OS benefit, but this trend does not presented at either PFS or ORR (median split, OS: high vs. low: 9.8 vs. 15.9, *p* = 0.33; PFS: high vs. low: 4.8 vs. 5.0, *p* = 0.69; ORR: high vs. low: 35% vs. 35%, *p* = 1; Supplementary Fig. 4H–J).

### Additional immune signatures analysis in predicting tumor response

**Fig. 5:**
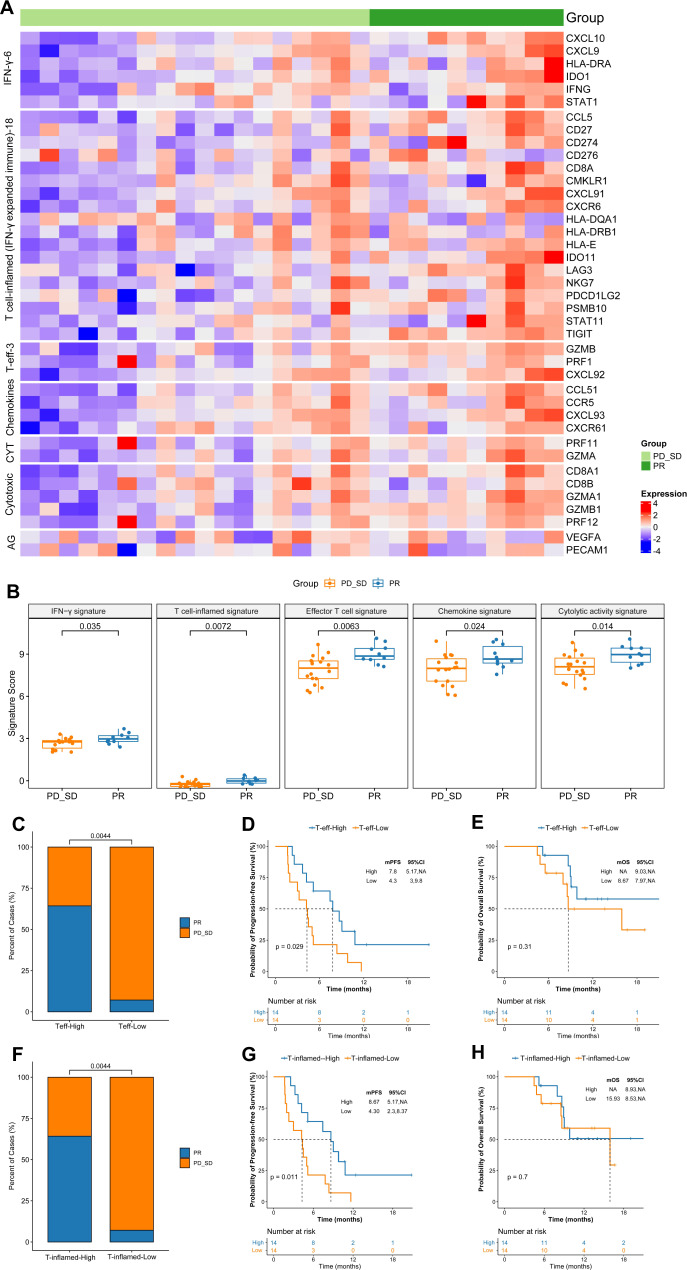
Additional immune signatures analysis in predicting tumor response. **A** The expression of 7 gene sets previously reported to be associated with response to immunotherapy between responders (*n* = 10) and non-responders (*n* = 18). **B** Five gene sets of prognostic value were differentially expressed between responders (*n* = 10) and non-responders (*n* = 18), box plots are indicated in terms of minima, maxima, centre, bounds of box and whiskers (interquartile range value), and percentile in the style of Tukey, Wilcoxon test was used to determine the statistical significance between subgroups. **C**–**E** Higher ORR and longer PFS were associated with higher expression of 3-gene effector T cell signature, 9 patients achieved PR in the higher expression of 3-gene effector T cell signature group (*n* = 14), and 1 patients achieved PR in the lower expression of 3-gene effector T cell signature group (*n* = 14). *P*-values were based on a two-sided log-rank test for survival analysis, Fisher’s exact test was used to determine statistical significance between the two groups. **F**–**H** Higher ORR and longer PFS were associated with higher expression of 18-gene inflamed T cell signature. 9 patients achieved PR in the high expression of 18-gene inflamed T cell signature group (*n* = 14), and 1 patients achieved PR in the low expression of 18-gene inflamed T cell signature group (*n* = 14). *P*-values were based on a two-sided log-rank test for survival analysis, Fisher’s exact test was used to determine the statistical significance between the two groups. Source data are provided as a Source Data file.

Furthermore, we assessed the expression levels of seven gene sets that were previously reported to be associated with response to immunotherapy (Fig. 5A). Notably, five gene sets of prognostic value were differentially expressed between responders and non-responders, namely 6-gene IFN-γ signature (*p* = 0.035), 18-gene inflamed T cell (IFN-γ expanded immune) signature (*p* = 0.007), 3-gene effector T cell signature (*p* = 0.006), chemokine signature (*p* = 0.024), and cytolytic activity signature (*p* = 0.014; Fig. 5B). In the above mentioned five differential gene expression signatures, higher ORR and longer PFS were associated with higher expression of 3-gene effector T cell signature (ORR: high vs. low: 64.2% vs. 7.1%, *p* = 0.004; median, PFS: high vs. low: 7.8 vs. 4.3 months, *p* = 0.02; Fig. 5C, D) and 18-gene inflamed T cell signature (ORR: high vs. low: 64.2% vs. 7.1%, *p* = 0.004; median, PFS: high vs. low: 8.6 vs. 4.3 months, *p* = 0.01; Fig. 5F, G). The correlation with OS did not reach statistical significance (Fig. 5E, H). No significant differences were observed in either survival outcome or clinical response in cytotoxic T cell score (*p* = 0.17) or angiogenesis score (*p* = 0.383).

As genetic alterations have been nominated to affect immune signatures and infiltration^20^, we next investigated the association between genetic alterations and the abundance of 14 predefined immune cells and immune signatures. We found that *ATM* mutation seems to be associated with lower mast cells score (*ATM* Mu vs. WT: 4.0 vs. 6.3, *p* = 0.062, Supplementary Fig. 5A); Patients with *BAP1* mutation presented lower Treg cells score (*BAP1* Mu vs. WT: 5.4 vs. 6.5, *p* = 0.038, Supplementary Fig. 5B) and Macrophages.M2 cells score (*BAP1* Mu vs. WT: 8.4 vs. 10.0, *p* = 0.081, Supplementary Fig. 5C). Similarly, HRR mutation was associated with lower Macrophages.M2 cells score (HRR Mu vs. WT: 9.2 vs. 10.2, *p* = 0.035, Supplementary Fig. 5D).

### TiME characteristics based on multiple immunofluorescence and clinical responses

**Fig. 6:**
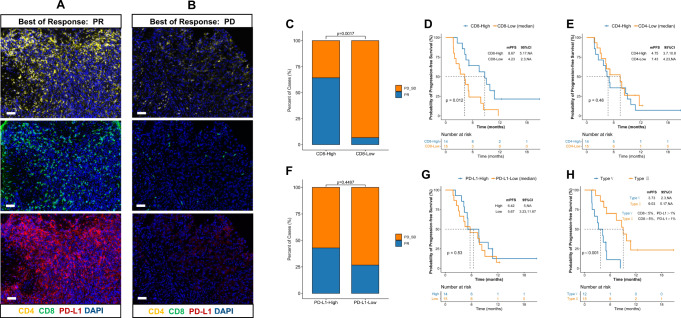
TiME characteristics by multiple immunofluorescence and clinical responses. **A**, **B** Multiple staining images of two typical patients. Multiple immunofluorescence staining were performed one time in 29 independent samples with similar results, responders (*n* = 11) and non-responders (*n* = 18), scale bar: 50 μm. **C** Significant differences with a high proportion of CD8^+^ T cells in responders, 9 patients achieved PR in the high CD8^+^ T cells group (*n* = 14), and 1 patients achieved PR in the low CD8^+^ T cells group (*n* = 15). Fisher’s exact test was used to determine the statistical significance between the two groups. **D** Longer PFS was associated with a higher proportion of CD8^+^ T cells, *P*-values were based on a two-sided log-rank test for survival analysis. **E** No significant difference was observed concerning CD4^+^ T cells and clinical response, *P*-values were based on a two-sided log-rank test for survival analysis. **F** Higher PD-L1 expression showed a better tumor response trend, 6 patients achieved PR in the PD-L1 high group (*n* = 14), and 4 patients achieved PR in the PD-L1 low group (*n* = 15). Fisher’s exact test was used to determine statistical significance between the two groups. **G** No significant differences were observed in survival outcome in the higher PD-L1 expression group, P-values were based on a two-sided log-rank test for survival analysis. **H** The combination of higher infiltrated CD8^+^ T cells with PD-L1 positive expression has a better benefit of PFS, P-values were based on a two-sided log-rank test for survival analysis.

We analyzed the immune cell markers (CD4, CD8, PD-L1) to further investigate the TiME using multiple immunofluorescence for estimating the spatial specificity subtypes of infiltrating immune cells (Fig. 6A, B). Analysis of 29 response-evaluable patients exhibited significant differences with a higher proportion of CD8+ T cells (median split, ≥ 5.46%) in responders than in non-responders (*p* = 0.0017, Fig. 6C), and longer PFS was associated with a higher proportion of CD8+ T cells (median, PFS: high vs. low: 8.6 vs. 4.2 months, *p* = 0.012, Fig. 6D). No significant difference was observed concerning CD4+ T cells and clinical response (Fig. 6E). Furthermore, high PD-L1 expression (median split, CPS ≥ 5%) showed a better tumor response trend (ORR: 46.67% vs. 26.67%, *p* = 0.44, Fig. 6F), whereas no significant differences were observed in survival outcomes in the high PD-L1 expression group (median, PFS: high vs. low: 5.0 vs. 6.4 months, *p* = 0.81, Fig. 6G). However, based on previous studies^14,15,21^, the combination of highly infiltrated CD8+ T cells (>5%) with PD-L1 (>1%) positive expression appeared to better predict the benefit of PFS with lower hazard ratio (HR) value than solely higher infiltrated CD8+ T cells (HR: 0.27 vs. 0.37, Fig. 6H). This observation was consistent with that of the four classical classifications of tumor immunity in the TiME, highlighting that both PD-L1 expression and immune infiltration are critical in improving the efficacy of cancer immunotherapy^23,24^.

## Discussion

GemCis has been the standard first-line treatment for unresectable or metastatic BTCs for decades. Although the ABC-02 and BT22 study demonstrated the clinical efficacy of GemCis^5,22^, alternative chemotherapy has not significantly improved the outcomes compared with that of GemCis^24–27^, which remains the preferred first-line systemic therapy in BTC. The TOPAZ-1 study exhibited that GemCis plus durvalumab is an effective and tolerated regimen for the first-line therapy of BTC^11^. Based on this information, immuno-chemotherapy has become a new option for patients with BTC. Our study was a phase II study which aims to investigate the efficacy and safety of sintilimab plus GemCis as the first-line treatment for treatment naïve BTC patients. A trend of benefit was observed with sintilimab and chemotherapy, especially toward OS and ORR, which provided clinical evidence on advanced BTC. Moreover, this study investigated the biomarkers that higher expression of IFN-γ-related signature and T cell-inflamed signature, along with HRR pathway gene alterations or loss-of-function mutations in chromatin remodeling genes may potentially predict the efficacy of immunocombination therapy on BTC.

In our study, 15.9 months of median OS and 36.7% of ORR emerged as a benefit tendency compared with chemotherapy and were comparable to TOPAZ-1^5,11^. Specifically, in a phase II study, the ORR was as high as 61.5% when nivolumab combined with GemCis was administered to treatment-naïve patients with BTC.^14^ The potent anti-tumor activity may be associated with a high dosage of GemCis. However, the OS was only 8.6 months, possibly due to the toxicity of the drugs. Moreover, the triplet therapy regimen (gemcitabine, nab-paclitaxel, and cisplatin) exhibited a high ORR of up to 45% but with a concerning safety profile^26^. A high risk of developing hematological toxicity and related complications was indicated, limiting its application in the elderly or patients with poor physical status. In our study, a promising result of OS and ORR did not significantly increase the incidence rate of SAEs, displaying manageable safety. It was important to note that we should be cautious when comparing data from other studies because survival data were immature until the data cut-off day and there were differences in the study designs and enrolled patient populations. It could be a possible reason that some early progressed patients may have contributed to a slightly shorter median PFS in our study because of per 6 weeks’ tumor response assessment, comparing with other trials whose response assessments were at per 12 weeks, such as ABC 02 study^5^. For the patients who achieved an objective response, the median duration of response was consistent with the immunotherapy characteristic of the objective response being sustainable once the drug takes effect^27^.

In our study, a treatment containing sintilimab combined with GemCis was well tolerated and within acceptable toxicity levels. The most frequently reported treatment-related grade 3 or above adverse events were hematological toxicities, which were widely perceived to be associated with cisplatin and gemcitabine. However, we observed a much higher incidence of grade 3 or above thrombocytopenia and a lower incidence of neutropenia in this study compared with that reported for GemCis chemotherapy^22–25^ and durvalumab plus GemCis in TOPAZ-1^11^. Moreover, one study reported a higher incidence of thrombocytopenia when nivolumab was combined with GemCis.^14^ On the whole, PD-1 inhibitor (sintilimab) plus GemCis demonstrate promising antitumor activity without additional toxicity, which could be another effective first-line therapy and provide further evidence for combine therapy in patients with ICC.

Although efforts have been made to reveal the genetic and immunological characteristics of BTC for determining its prognostic value, progress is slow and the findings may be difficult for widespread application. TMB, as a widely used immunotherapy predictive biomarker, was not associated with the clinical benefit of sintilimab combined with GemCis in our trial. This observation is consistent with that in KEYNOTE 407 and KEYNOTE 189 studies for NSCLC^28,29^ and in toripalimab plus gemcitabine and S-1 (GS)^15^, nivolumab plus GemCis^14^, and camrelizumab plus gemcitabine and oxaliplatin (GEMOX) trials^13^ for advanced BTCs. These findings highlighted that TMB may not be an effective biomarker for predicting the clinical benefit of PD-1/PD-L1 antibodies plus chemotherapy. In addition to TMB, positive PD-L1 expression was unreliable in determining the predictive value of immuno-chemotherapy for BTCs. In our trial, high PD-L1 expression showed a better tumor response trend but no significant difference in the survival outcome. Compared with previous studies on BTCs, the predictive value of positive PD-L1 exhibited not only similarities but also discrepancies. For instance, analyses of patients with BTC treated with nivolumab plus GC indicated that the PD-L1 expression level (Dako 22C3, combined positive score (CPS) > 1%) could not be used as a biomarker for predicting clinical response.^14^ In contrast, in the toripalimab plus GS trial for patients with BTC, positive PD-L1 expression (Dako 22C3, CPS > 1%) led to a statistically prolonged PFS.^15^ Furthermore, PD-L1 expression (Ventana SP263, tumor proportion score >1%) in tumor cells, rather than in tumor-infiltrating immune cells, was associated with response in patients with BTC treated with camrelizumab plus GEMOX.^13^ Thus far, there is no consensus on antibody selection for detecting PD-L1 expression in BTCs. These seemingly contradictory findings may be due to the inconsistency of antibodies or detection platforms used in the respective studies. Additionally, the immunofluorescence method for detecting the expression of PD-L1 in our study may have significantly increased the sensitivity of detection, which is one of the reasons for the higher expression of positive PD-L1 observed in our study than that in other studies. Moreover, the heterogeneity and complexity of BTC may also be key factors for the heterogeneous results.

Homologous recombination deficiency (HRD) prevents DNA-damaging agents such as platinum-based chemotherapy from repairing gene damage, disrupts the ability of cells to undergo homologous recombination, and further causes gene instability^30^. Importantly, in our findings, HRR pathway gene mutations were associated with clinical benefit in survival outcome and tumor response, which might be because dysfunctional HRR function is a determinant of sensitivity to platinum chemotherapy. Previous studies have demonstrated that HRD, in addition to being a predictor of PFS and OS in patients who would benefit from platinum-based chemotherapy, is also a major indicator for platinum-sensitive drugs in patients with ovarian^31,32^ and pancreatic cancer^33,34^. Therefore, to some extent, the choice of maintenance therapy, including mono-immunotherapy or chemotherapy, should be fully considered based on HRD status in future clinical practice.

Tumor-infiltrating lymphocytes (TILs) have been proposed as a crucial prognostic indicator, and the TILs density has great predictive power for survival in solid cancers^35,36^. Reportedly, several TIL subtypes in TiME, such as CD3^+^ cells, are potentially related to combination therapy using nivolumab plus GC in patients with advanced BTCs^14^. The prognostic impact of tumor-infiltrating immune cells on BTCs was also investigated. The number of tumor-infiltrating Foxp3^+^ regulatory T lymphocytes and CD4^+^ T lymphocytes were tumor grade-independent prognosticators^37^. One of the most significant findings in our study was that high levels of tumor-infiltrating MCs are associated with significantly shorter survival outcomes and ORRs. In addition to their traditional roles in allergy and host defense, MCs are key immunomodulatory cells that may play a vital and under-explored, but therapeutically targeted role, in tumor immunity^38^. In previous studies, MCs have been shown to play a tumor-promoting role in regulating melanoma tumor angiogenesis and tumor growth. The presence of tumor-infiltrating mast cells are associated with downmodulation of HLA-class I on tumor cells, lack of CD8+ T cells in these areas, and in effective tumor killing and eventual immune escape after anti-PD-1 therapy^39^. Transcriptome analysis in our study revealed that MCs produced IL-4 expression, intratumoral myeloid-derived suppressor cells were associated with CD244 expression, and some carcinoembryonic antigen genes were upregulated in non-responders. CIBERSORT analysis of RNASeq data set from MD Anderson trial (pre-therapy patients, *n* = 23) revealed higher mast cell levels in anti-PD-1 therapy non-responder population^39^. Consistent with our findings, there was an increased abundance of mast cells in non-responder patients when compared to patients responding to anti-PD-1 therapy.

Activated MCs are thought to release a wide range of bioactive molecules that affect invasion, tumor-associated angiogenesis, and immune cell activity, leading to tumor growth and metastasis^40^. For instance, a recent cholangiocarcinoma study reported tumor infiltrating mast cells participate in the progression and metastasis via c-kit/stem cell factor-dependent signaling^41^. Similar findings that higher pre-treatment mast cell infiltration is significantly associated with poor responses to pre-surgical chemotherapy in an aggressive form of localized breast cancer^42^, and higher mast cell tumor infiltration predicts poor responses to anti-PD-1 ICB in melanoma^39^. There are, of course, studies with seemingly contradictory conclusions, Xiaobo Bo et al. demonstrated increased efficacy of adjuvant gemcitabine-based chemotherapy for patients whose BTCs had a higher mast cell infiltration^43^. It’s worth noting that some notable features in that study, including tumor type, cancer stages, drug intervention, the location of the mast cells within the tumor, and the detection methods were different from our study. In addition to the dual characteristics of mast cells, these may be the reason for the different results in our two studies. Thus, although there is growing evidence that MCs continuously infiltrate tumors, however, it is unclear whether their role in tumor immunity is protective or harmful and to what extent this role is determined by local inflammatory TiME^44^. Consequently, their role in oncology remains opaque. To the best of our knowledge, we report the adverse predictive value of tumor-infiltrating MCs in immunotherapy combined with chemotherapy for BTCs. Therapeutics aimed at targeting MCs were determined to have the potential in BTCs for precision immunotherapy.

The TiME mainly comprised cellular and non-cellular components that play an important role in anti-tumor activity^45,46^. Relying on the high-throughput transcript analysis, we attained a deep understanding of the predominant TiME characteristics of immuno-chemotherapy in BTCs. In our representative findings, we identified that an 18-gene T cell-inflamed signature was associated with longer PFS and better clinical response. T cell-inflamed signature is known to characterize the adaptive Th1 and cytotoxic CD8^+^ T cell responses, including IFN-γ signaling, cytolysis activity, antigen presentation, T cell transport, and apparent inhibitory mechanisms in T cell homeostasis^47^. Furthermore, it describes the significant TiME signature of BTC immuno-chemotherapy. These characteristics also suggest that CD8^+^ T lymphocytes and T-cell active chemokines are co-regulated in tumors as well as in T-cell counter suppressor molecules such as PD-1/PD-L1 and IDO1^48–50^. Consistent with the features of immune superiority, interferon-induced-related genes, chemokines, and immune checkpoint targets were found to be upregulated in the responders. Moreover, the combination of highly infiltrated CD8^+^ T cells with positive PD-L1 expression appeared to better predict the benefit of PFS with lower HR value than solely highly infiltrated CD8^+^ T cells. This observation was consistent with that of the four classifications of tumor immunity in the microenvironment, highlighting that both PD-L1 expression and immune infiltration are critical in improving the efficacy of cancer immunotherapy.

Owing to the limited number of clinical studies, our study had some limitations. As it is a single-center study and intrahepatic cholangiocarcinoma is the main type of BTCs in this ward at the center, the patients enrolled were relatively restricted in diversity of BTC subgroups. Thus, the significance of extrahepatic cholangiocarcinoma and gallbladder carcinoma is limited. A larger randomized and multi-center trial is warranted to verify the study results. Besides, while we formulated the standard of the specimens, since the heterogeneity and purity difference of biopsy tumors, and the exploration on bulk-tumor level have inherent limitations in providing precise information, these are the unavoidable methodological limitations in our study. Not only that, biomarker analyses in our study were retrospective and hypothesis-generating in limited sample size, and therefore the results should be interpreted with caution. In summary, our study suggested that sintilimab plus GemCis delivered promising antitumor efficacy and an acceptable safety profile in advanced BTCs. Moreover, the genetic and TiME characteristics were deeply investigated to establish effective biomarkers for predicting the clinical response. Our findings provide a potential treatment option and a certain basis for further study of this regimen in BTCs.

## Methods

### Study design and participants

This was a single-armed, open-labeled, phase II, prospective study (ChiCTR2000036652), preregistered on 24th Aug, 2020. The study was conducted according to the guidelines dictated by the Helsinki Declaration and international standards of good clinical practice. The Ethics Committee of Eastern Hepatobiliary Surgery Hospital approved the protocol and any protocol amendments. All the enrolled patients provided a written informed consent form. In this study, we enrolled 30 patients with advanced BTC receiving GemCis plus sintilimab between August 2020 and May 2022 in the Shanghai Eastern Hepatobiliary Surgery Hospital. Newly diagnosed 18–75-year-old patients with histologically and cytologically confirmed BTC were eligible for inclusion. Furthermore, patients with at least one measurable lesion as the target lesion according to RECIST V.1.1 and an Eastern Cooperative Oncology Group performance status of 0–2 were eligible for inclusion. The exclusion criteria for the patients were as follows: the presence of secondary malignancies or other types of tumors with metastasis to the brain or meninges within 3 years before study initiation and incidence of concomitant diseases that, in the investigator’s judgment, may seriously endanger their safety or interfere with the completion of the study.

### Procedures

Gemcitabine (1000 mg/m²) and cisplatin (25 mg/m²) were administered to the patients on days 1 and 8, respectively, per 21-day cycle, with a total of 6–8 cycles of chemotherapy. Sintilimab (200 mg) was intravenously administered on day 1 of each 21-day cycle. This combination regimen was sustained until disease progression, intolerable toxicity, or completion of 6–8 cycles of the treatment. The maintenance treatment with sintilimab was administered for 2 years to patients who completed 6–8 cycles (determined by the treating physician and the patient) of the combination therapy. During this treatment period, any kind of dose modification of sintilimab was not allowed.

Patients could withdraw from this trial at any point without disrupting their standard therapy or harming their chances of potential participation in other research studies. Investigators could terminate the participation of patients if they had reason to believe that further treatment would be harmful to their well-being. Patients accepted evaluation of efficacy per 6 weeks, examination of blood routine, liver and kidney function, levels of serum electrolytes coagulation, tumor indicators, and immune-related indicators were reviewed simultaneously. Furthermore, they were evaluated for safety profiles during each visit. Detailed study protocol and statistical analysis plan were provided in the supplementary materials.

### Endpoints

The primary endpoint was OS, which indicates the day from the first dose of GemCis or sintilimab to death from any cause. Secondary endpoints included progression-free survival (PFS; the day from the first dose to disease progression or death), ORR, the proportion of complete response rate plus partial response (PR) rate under computerized tomography, and disease control rate [DCR; the proportion of patients who achieved CR plus PR and Supplementary Table disease (SD)]. As to render safety, adverse events (AEs) in the entire study process were reported as per CTCAE V.5.0. Imaging and pathology were confirmed by two clinical specialists. Investigators evaluated the responses based on RECIST V.1.1. Toxicity profile included the events occurring 30 days after the end of therapy in all patients. Multiomics biomarkers associated with clinical response were assessed as an exploratory objective.

### Sample collection

All biomarker investigations in this study were based on baseline pre-treatment biopsies, no other biopsies were obtained during the course of the study. In view of ethical compliance and safety, specimens from core needle biopsies were obtained in two or three directions in the primary cholangiocarcinoma tumor to reducing the effect of tumor heterogeneity. In terms of tumor purity, each specimen was hand-reviewed by a pathologist to ensure it was suitable for sequencing and we kept only specimens with ≥40% tumor purity. For tumors purity below this threshold, laser-capture microdissection was used to mark tumor areas on tissue sections attached to glass slides to improve tumor purity.

### DNA extraction and library preparation

A minimum of 20% tumor content was required needed for clinical formalin-fixed paraffin-embedded (FFPE) samples used for genomic evaluation. Genomic DNA (gDNA) of formalin-fixed and parrffin-embedded (FFPE) tissues was extracted using Genomic DNA Tissue Extraction Kit (Concert®),and matched peripheral blood was extracted using Magnetic Universal Genomic DNA Kit (TIANGEN). NGS tests targeting Whole exome Hyb Panel were performed at Simceredx company (Nanjing,China), and following manufacturers’ instructions. In brief, 200 ng gDNA was sheared into 200~300 bp by enzymatic fragmentation kit. Indexed paired-end adaptors for Illumina platform were synthesized by Integrated DNA Technologies (IDT). End repair, A-tailing, and adaptor ligation of sheared DNA were performed with the reagents from KAPA Hyper DNA Library Prep kit (Roche Diagnostics). Unligated adaptors were removed by the size selection function of Agencourt AMPure XP beads (Beckman Coulter) and the ligation products were PCR amplified to form a prelibrary for hybridization.

### Library sequencing and bioinformatics analysis

Prepared DNA libraries were sequenced on Illumina NovaSeq 6000 platform (Illumina, San Diego, CA) and generate 150 bp paired-end reads. The principle of sequencing is Sequencing by Synthesis. The fastp tool (V.2.20.0) was used for adapter pruning and to filter low-quality sequencing reads^51^. Cleaned reads were aligned to the human reference genome (hg19) using the BWA-mem algorithm^52^. Somatic mutations including point mutations, small insertions, and deletions were identified and annotated using VarDict and InterVar, respectively^53,54^. We screened for germline variations using the internal database. Copy number variation involved amplification and deletion were identified by CNVkit^55^. TMB measurements considered only single nucleotide variants and insertions and deletions in the coding region. TMB high was defined as the top 50% value. In term of MSI status analysis, all 30 samples were tested for MSI by a NGS-based custom panel, of which 25 patients with sufficient specimens remaining were re-evaluated by IHC. In term of the custom pipeline for MSI status analysis, only mononucleotide repeat loci with repeat length exceeds 10, and sequence depth greater than 50 were selected. Based on hundreds of PCR-confirmed samples, the prior probability of MSI-H-pattern reads at each loci was calculated. For each sample, we than use binomial distribution to calculate the probability of MSI-H pattern reads for each loci, If the probability is significantly low (≤0.001), we would define the loci as MSI-H in this sample. Finally, samples with MSI scores (the percentage of MSI-H loci in this sample) ≥20 were considered to be microsatellite unSupplementary Table, while the rest were considered microsatellite Supplementary Table.

### Transcriptional profiling and analysis

Total RNA was isolated from FFPE slices using the Qiagen RNeasy FFPE Kit, followed by hybridization of 100 ng RNA to a version of the NanoString PanCancer code set for reading on the nCounter platform. The expression levels of 289 immune-related genes, including housekeeping genes, are listed in Supplementary Table 3. The housekeeping genes were employed to normalize the expression values, as recommended by the manufacturer, using nSolver 2.6 software. Differentially expressed genes were selected through the “DEseq2” software package, with log2 |fold change|> 1 and false discovery rate <0.05. Heatmaps of differentially expressed genes were created using the “ComplexHeatmap” package. According to the manufacturer’s specification, the genes were divided into 14 immune cell types: T cells, B cells, mast cells (MCs), dendritic cells, macrophages, neutrophils, cytotoxic cells, exhausted CD8 cells, NK-CD56 cells, CD8 T cells, CD45 cells, Th1 cells, NK cells, and Treg cells (Supplementary Table 4). Metagene scores were calculated based on the geometric mean of expression levels of the member genes. In term of HRD transcriptomic signature, genes (*ATM*, *BLM*, *BRCA1/2*, *BRIP1*, *NBN* and *RAD51*) that overlapped the HRR pathway within 289 genes in the customized panel were included. Furthermore, we studied seven previously published prognostic and immunotherapy response gene sets and their metagene scores using the previously described methods (Supplementary Table 5). Nanostring normalized genes expression data analyzed in this study was supplied as Supplementary Data 1.

### Multiplexed immunofluorescence

Leica Bond RX was used for multiplex immunofluorescence staining according to the manufacturer’s instructions. The following primary antibodies were used for immunostaining: anti-CD4 (Ventana, SP35, 1:100), anti-CD8 (Ventana, SP57, 1:400), and anti-PD-L1 (Ventana, SP263, 1:250) antibodies. We used 4′,6-diamidino-2-phenylindole (Sigma) to stain the nuclei.

### Statistical analysis

This was a single-arm study, and no randomization was used. To determine the sample size for this clinical trial, overall survival (OS) with standard of care chemotherapy (Gem/Cis) as the historical control was assumed to be 9.5 months based on previously reported data of Asian population^56^ and the notable features of the high proportion of ICC patients in our center. The addition of sintilimab to chemotherapy would expect to improve the OS to 16.0 months. Given an accrual period of 24 months, a maximum follow-up time of 48 months, at the significance level of 0.05, to achieve the power of 0.8, the number of events required is 23. Equivalently, a sample size of 30 is needed^57^. The sample size result is based on a one-sided test with exponential assumption for survival time.

Statistical analyses and graph illustration were performed using SAS 9.4 (SAS Institute, Cary, NC, USA) and R 4.1.3. Survival was analyzed using Kaplan–Meier curves and log-rank test, and values of *p* < 0.05 were considered to be statistically significant in the remaining statistical analyses. We calculated 95% CI using the exact Clopper–Pearson method. Safety outcomes were analyzed in patients who received one of the aforementioned doses of the study regimen.

### Reporting summary

Further information on research design is available in the Nature Portfolio Reporting Summary linked to this article.

## Supplementary information

Supplementary Information

Description of Additional Supplementary Files

Supplementary Data 1

Reporting Summary

## Data Availability

The genomic raw sequencing data generated in this study have been deposited in the Genome Sequence Archive in National Genomics Data Center, China National Center for Bioinformation under accession code HRA003603. The raw sequencing data are under restricted access due to data privacy laws and are available upon request for 1 year. Data are available on request sharing by sending requests to the corresponding author Zhen-gang Yuan (yuanzg@smmu.edu.cn), which will need the approval of the institutional ethical committees. Access can be obtained by completing the application form via GSA-Human System. Clinical data are not publicly available due to involving patient privacy, but can be accessed from the corresponding author, upon request for 3 years; individual de-identified patient data will be shared for clinical study analyses. The study protocol is provided in the Supplementary Information file. Source data are provided with this paper. Nanostring normalized gene expression data analyzed are available as Supplementary Data 1. The remaining data are available in the Article, Supplementary Information, or Source Data file. Source data are provided with this paper.

## Source data

Source Data
