# Supplementary material for: Clinical and biomarker analyses of sintilimab plus gemcitabine and cisplatin as first-line treatment for patients with advanced biliary tract cancer
Source: Nat Commun. 2023 Mar 11;14:1340. doi: 10.1038/s41467-023-37030-w (PMC10008621; doi:10.1038/s41467-023-37030-w)
Supplement: Supplementary file 1 — Supplementary Information [file 41467_2023_37030_MOESM1_ESM.pdf]

# Contents

1. Supplementary Figure 1: Part of the IHC staining results for MSI testing.
2. Supplementary Figure 2: Association between gene alterations and clinical response.
3. Supplementary Figure 3: GO enrichment and KEGG pathways analysis of differential expression genes.
4. Supplementary Figure 4: Additional immune cell analyses in responders and non-responders.
5. Supplementary Figure 5: The association between genetic alterations and the abundance of 14 predefined immune cells and immune signatures.
6. Supplementary Table 1: The distribution of somatic gene alteration and HRR pathway genes associated with survival outcome.
7. Supplementary Table 2: Gene expression levels (median) associated with survival outcome.
8. Supplementary Table 3: 289 immune-related genes, including housekeeping genes list.
9. Supplementary Table 4: The genes list of 14 immune cell types.
10. Supplementary Table 5: Signatures/Scores gene list and respective references.
11. Supplementary Note: Study protocol and SAP.

# Supplementary Figure 1

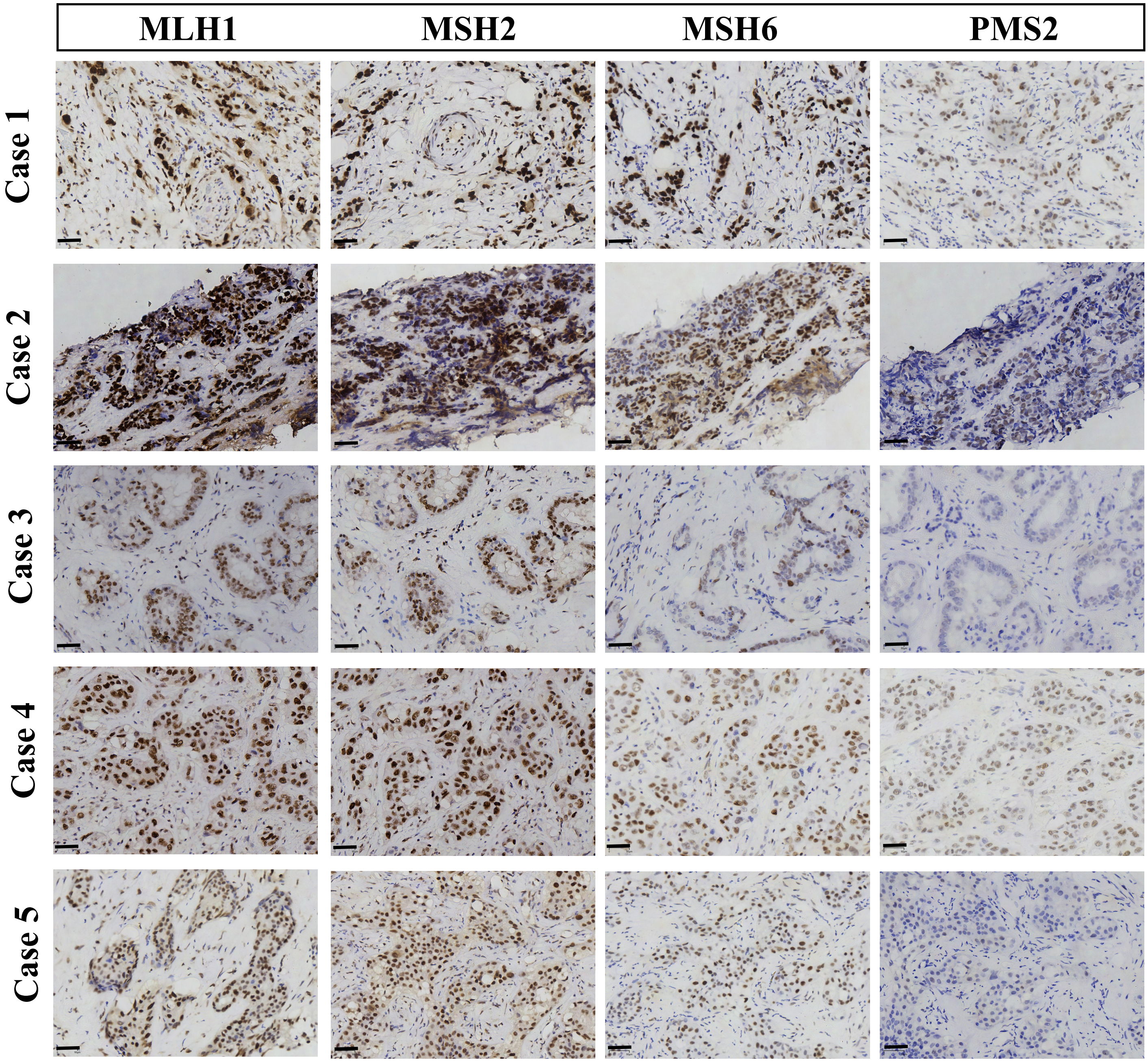


**Supplementary Figure 1:** Part of the IHC staining results for MSI testing. IHC staining were performed one time in 25 independent samples with similar results. Scale bar: 50μm.

# Supplementary Figure 2

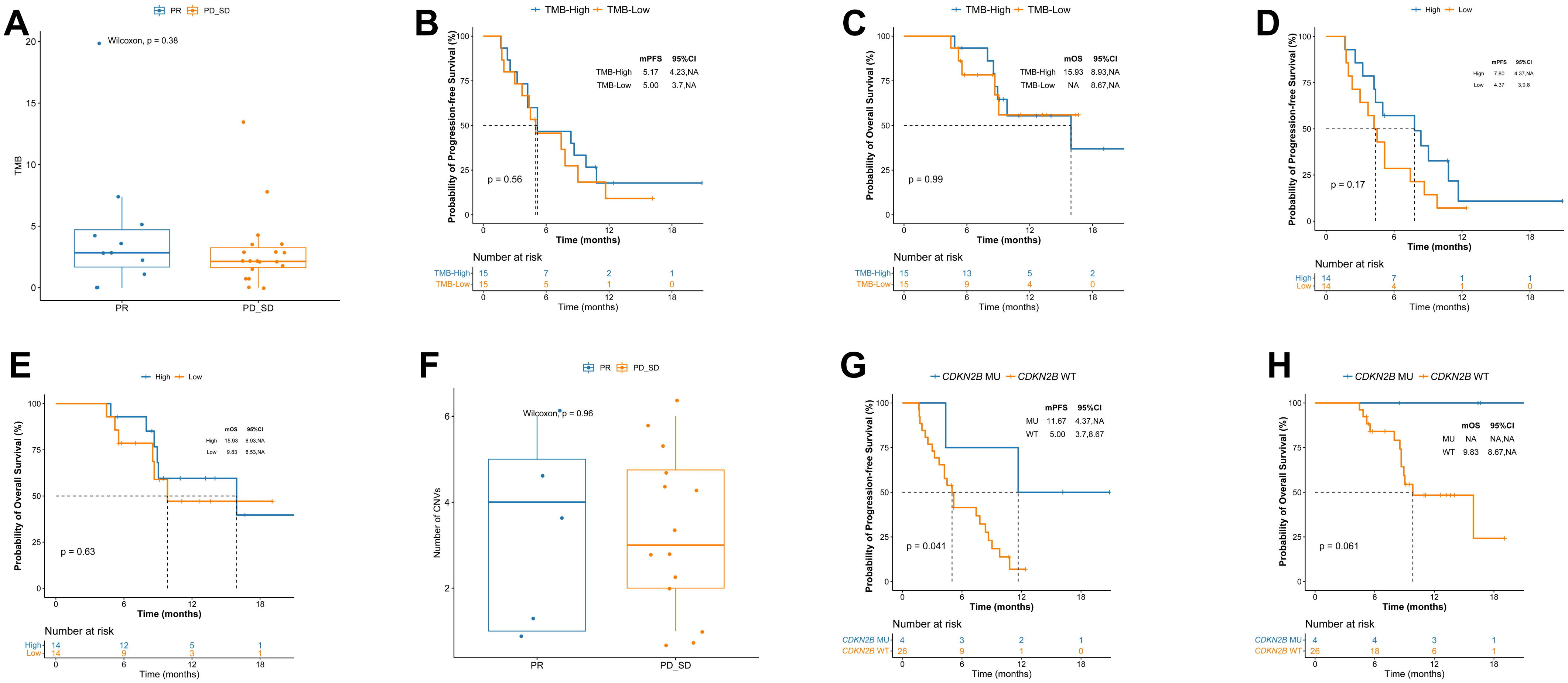


**Supplementary Figure 2:** Association between gene alterations and clinical response. (A) The median TMB in responders (n=11) and non-responders (n=19), box plots are indicated in terms of minima, maxima, centre, bounds of box and whiskers (interquartile range value), and percentile in the style of Tukey, Wilcoxon test was used to determine the statistical significance between subgroups. (B, C) No association was observed between TMB-high and low (median split) in PFS or OS, P-values were based on a two-sided log-rank test for survival analysis. (D) high HRD transcriptomic signature score tended to have better PFS compared to the low group, P-values were based on a two-sided log-rank test for survival analysis. (E) No OS benefit was found in high HRD transcriptomic signature score compared to the low group, P-values were based on a two-sided log-rank test for survival analysis. (F) No association was observed between the total number of copy number variants in responders (n=5) and non-responders (n=14), box plots are indicated in terms of minima, maxima, centre, bounds of box and whiskers (interquartile range value), and percentile in the style of Tukey, Wilcoxon test was used to determine the statistical significance between subgroups. (G, H) *CDKN2B* mutation showed longer survival trend in the association with PFS and OS, P-values were based on a two-sided log-rank test for survival analysis.

# Supplementary Figure 3

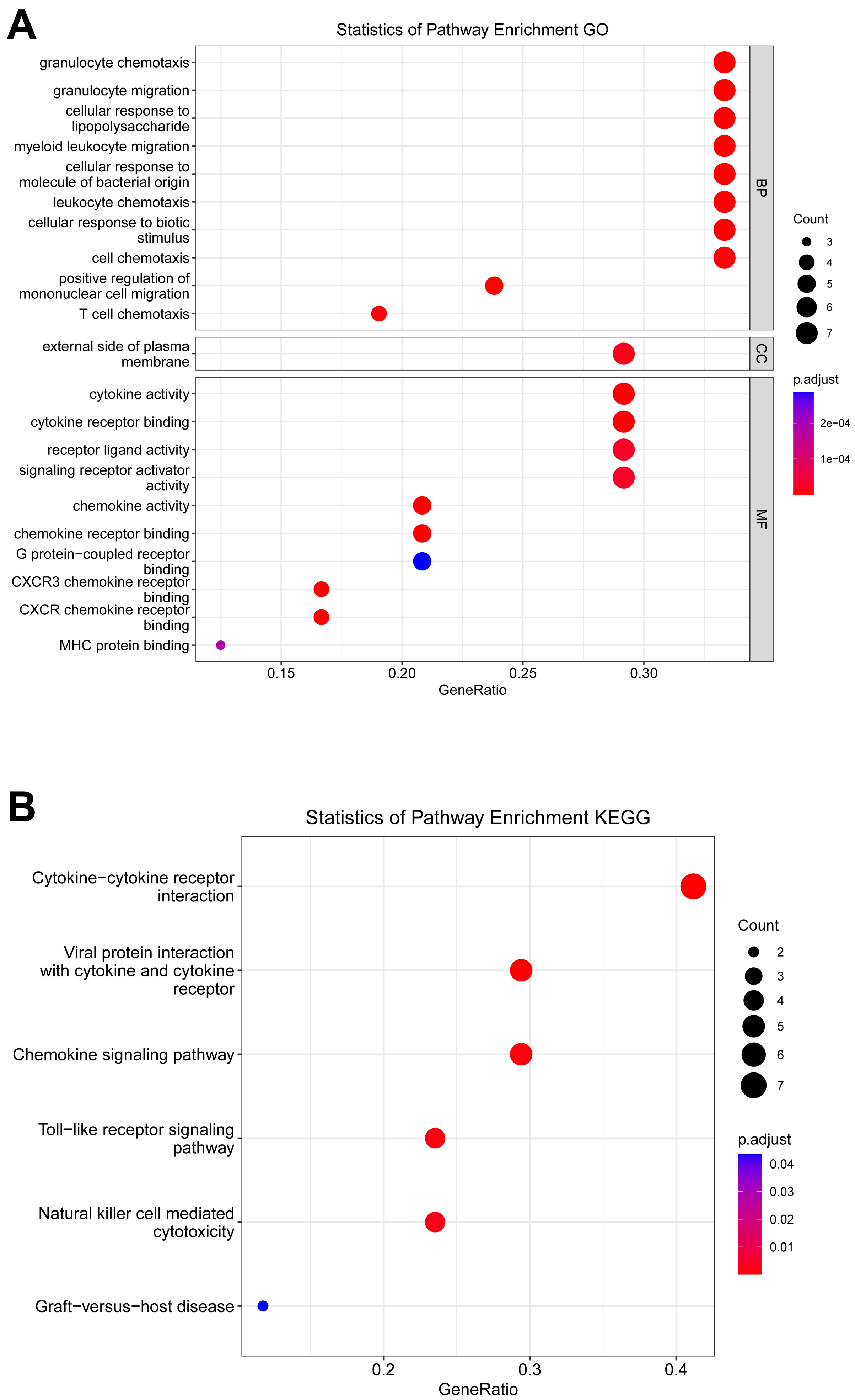


**Supplementary Figure 3:** GO enrichment and KEGG pathways analysis were performed to identify the molecular function of differential expression genes. (A) GO enrichment analysis of differential expression genes. (B) KEGG enrichment analysis of differential expression genes.

# Supplementary Figure 4

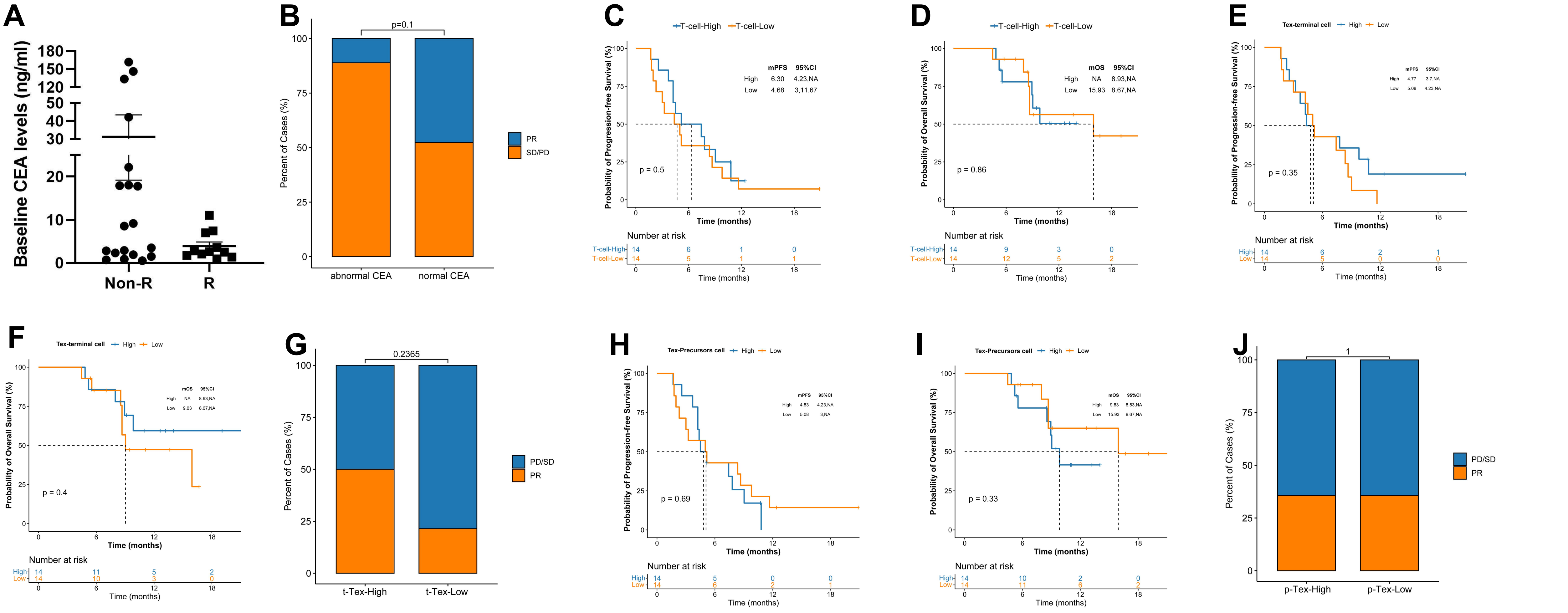


**Supplementary Figure 4:** Additional immune cell profile analyses in responders and non-responders. (A) baseline CEA levels in non-responders (n=19) were significantly higher than those in responders (n=11), data are presented as mean values +/- SEM, Fisher's exact test was used to determine statistical significance between the two groups. (B) patients achieved an objective response in the abnormal CEA group (≥10 ng/ml) and in the normal (≤10 ng/ml) CEA group, one patients (1/9, 11.1%) achieved an objective response in the abnormal CEA group, 10 of 21 patients achieved an objective response in the normal CEA group. Fisher's exact test was used to determine statistical significance between the two groups. (C, D) No significant differences were observed in survival outcome in the higher T-cell score group. (E-G) No significant difference was observed concerning the degree of infiltration of $Tex^{term}$ cells and clinical response, 7 patients achieved PR in the $Tex^{term}$ high group (n=14), and 3 patients achieved PR in the $Tex^{term}$ low group (n=14). Fisher's exact test was used to determine statistical significance between the two groups for ORR, P-values were based on a two-sided log-rank test for survival analysis. (H-J) No significant difference was observed concerning the degree of infiltration of $Tex^{pro}$ cells and clinical response, 5 patients achieved PR in the $Tex^{pro}$ high group (n=14), and 5 patients achieved PR in the $Tex^{pro}$ low group (n=14). Fisher's exact test was used to determine statistical significance between the two groups for ORR, P-values were based on a two-sided log-rank test for survival analysis.

# Supplementary Figure 5

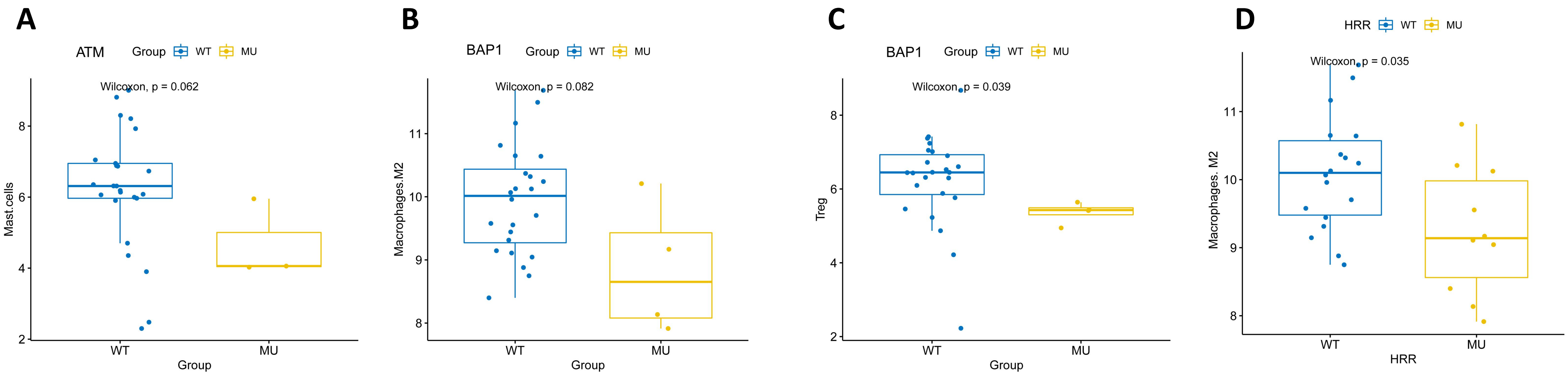


**Supplementary Figure 5:** The association between genetic alterations and the abundance of 14 predefined immune cells and immune signatures. (A) *ATM* mutation (n=3) seems to be associated with lower mast cells score compared with wild type (n=25), Wilcoxon test was used to determine the statistical significance between subgroups. (B, C) Patients with *BAP1* mutation (n=4) presented lower Treg cells score and Macrophages.M2 cells score compared with wild type (n=24), Wilcoxon test was used to determine the statistical significance between subgroups. (D) HRR mutation (n=10) was associated with lower Macrophages.M2 cells score compared with wild type (n=18), Wilcoxon test was used to determine the statistical significance between subgroups. All Box plots are indicated in terms of minima, maxima, centre, bounds of box and whiskers (interquartile range value), and percentile in the style of Tukey.

**Supplementary Table 1: The distribution of gene alteration associated with survival outcome**

| gene | KM_pvalue (PFS) | HR (PFS) | KM_pvalue (OS) | HR (OS) |
|---|---|---|---|---|
| ARID1A | 0.286 | 0.532 | 0.754 | 0.795 |
| ATM | 0.25 | 0.443 | 0.1 | 0 |
| BAP1 | 0.066 | 0.426 | 0.04 | 0 |
| BRD4 | 0.756 | 1.208 | 0.644 | 0.622 |
| BTG2 | 0.092 | 2.624 | 0.406 | 1.871 |
| CDK4 | 0.669 | 0.733 | 0.617 | 1.464 |
| CDK6 | 0.645 | 0.716 | 0.275 | 0 |
| CDKN2A | 0.182 | 0.529 | 0.869 | 0.899 |
| CDKN2B | 0.041 | 0.269 | 0.061 | 0 |
| DDR2 | 0.957 | 0.968 | 0.944 | 0.948 |
| ERBB2 | 0.328 | 1.788 | 0.331 | 0 |
| ERBB4 | 0.152 | 2.31 | 0.047 | 4.019 |
| FAT3 | 0.037 | 0.167 | 0.504 | 0.508 |
| IDH1 | 0.316 | 0.378 | 0.356 | 0 |
| KRAS | 0.954 | 1.024 | 0.063 | 2.809 |
| LRP1B | 0.034 | 3.309 | 0.846 | 0.819 |
| MCL1 | 0.26 | 1.821 | 0.954 | 1.045 |
| MUC16 | 0.135 | 1.865 | 0.575 | 1.402 |
| MYC | 0.06 | 2.219 | 0.466 | 1.551 |
| TERT | 0.193 | 2.146 | 0.837 | 1.235 |
| TP53 | 0.097 | 1.914 | 0.476 | 1.497 |
| ZNF217 | 0.005 | 4.57 | 0.259 | 0 |

| gene | KM_pvalue (PFS) | HR (PFS) | KM_pvalue (OS) | HR (OS) |
|---|---|---|---|---|
| BRAC1/2 | 0.26 | 2.0 | 0.21 | 0.31 |
| PALB2 | 0.80 | 1.2 | 0.45 | 0.34 |
| BLM | 0.94 | 0.94 | 0.36 | 0.33 |
| RECQL4 | 0.89 | 0.87 | 0.26 | 2.97 |

**Supplementary Table 1:** The distribution of somatic gene alteration and HRR pathway genes associated with survival outcome, P-values were based on a two-sided log-rank test. Genes with more than 10% mutation rate were included in the above table. HRR pathway genes mutation were included in the below table.

**Supplementary Table 2：gene expression levels (median) associated with survival outcome**

| gene | PFS_km_pvalue | OS_km_pvalue |
|---|---|---|
| SNAI1 | 0.003 | |
| FAS | 0.0063 | |
| IL1B | 0.031 | |
| IL2RG | 0.0231 | |
| TIE1 | 0.0077 | 0.0264 |
| CCL13 | 0.015 | |
| CCL22 | 0.0377 | |
| CCL5 | 0.0122 | 0.039 |
| CCR4 | 0.0445 | |
| CXCL1 | 0.0365 | |
| CXCL10 | 0.0051 | |
| CXCL11 | 0.0122 | |
| CXCR3 | 0.0282 | |
| CD274 | 0.0249 | |
| LAG3 | 0.0008 | |
| SIGLEC5 | 0.0096 | |
| S100A9 | 0.0409 | 0.0287 |
| CD1C | 0.0206 | |
| MLANA | 0.0078 | |
| TAP1 | 0.0196 | |
| HERC6 | 0.0459 | 0.0152 |
| PSMB9 | 0.0159 | |
| CPA3 | 0.0042 | |
| MS4A2 | 0.0316 | |
| TPSAB1 | 0.004 | 0.0415 |
| CEACAM3 | 0.0075 | |
| CD80 | | 0.031 |
| CD8A | 0.0024 | |
| CD40LG | 0.0036 | |
| GZMB | 0.0099 | |
| KLRD1 | 0.0316 | |
| KLRK1 | | 0.0232 |
| PRF1 | 0.0204 | |
| STAT1 | | 0.0346 |
| MSH2 | | 0.0252 |
| NOS2 | 0.0144 | |
| RAD51 | | 0.0165 |
| PTPRC | 0.0119 | |
| BLK | | 0.0303 |

**Supplementary Table 2：** Gene expression levels (median) associated with survival outcome, P-values were based on a two-sided log-rank test.

**Supplementary Table 3: TME 289 gene list**

| | | | | | |
|---|---|---|---|---|---|
| ABCF1 | CD44 | FCGR1A | IL21R | NFKBIA | TIE1 |
| ADM | CD47 | FCGR2B | IL2RA | NKG7 | TIGIT |
| ADORA2A | CD48 | FCRL2 | IL2RB | NOS2 | TLR3 |
| AKT1 | CD6 | FGF13 | IL2RG | NT5E | TLR7 |
| ANGPT2 | CD68 | FOXP3 | IL4 | OAS1 | TLR8 |
| ARG1 | CD69 | FPR1 | IL6 | OAS2 | TLR9 |
| ATM | CD70 | FUT4 | IL7R | OAS3 | TNF |
| AXL | CD74 | G6PD | IRF1 | PDCD1 | TNFRSF14 |
| BCL2 | CD79A | GBP1 | IRF4 | PDCD1LG2 | TNFRSF17 |
| BIRC5 | CD79B | GNLY | IRF9 | PDGFA | TNFRSF18 |
| BLK | CD80 | GUSB | ISG15 | PDGFB | TNFRSF1A |
| BLM | CD84 | GZMA | ITGA1 | PECAM1 | TNFRSF1B |
| BRCA1 | CD86 | GZMB | ITGAE | PIK3CA | TNFRSF4 |
| BRCA2 | CD8A | GZMH | ITGAL | PIK3CD | TNFRSF9 |
| BRIP1 | CD8B | GZMK | ITGAM | PMS2 | TNFSF10 |
| BTLA | CDKN2A | HAVCR2 | ITGAX | PNOC | TNFSF13B |
| C1QA | CEACAM3 | HDC | ITGB2 | POLR2A | TNFSF18 |
| C1QB | CMKLR1 | HERC6 | KIR2DL3 | PRF1 | TNFSF4 |
| CCL13 | CPA3 | HIF1A | KIR3DL1 | PSMB10 | TNFSF9 |
| CCL18 | CSF1R | HLA-DMA | KIR3DL2 | PSMB9 | TRAT1 |
| CCL2 | CSF2 | HLA-DMB | KLRB1 | PTEN | TWIST1 |
| CCL20 | CSF2RB | HLA-DOA | KLRD1 | PTGER4 | VCAM1 |
| CCL21 | CSF3R | HLA-DOB | KLRK1 | PTGS2 | VEGFA |
| CCL22 | CTAG1B | HLA-DPA1 | LAG3 | PTPN11 | VTCN1 |
| CCL4 | CTLA4 | HLA-DQA2 | LCK | PTPRC | ZAP70 |
| CCL5 | CTSS | HLA-DRA | LILRB2 | PVR | ZEB1 |
| CCL7 | CTSW | HSD11B1 | LY9 | RAD51 | CXCL2 |
| CCND1 | CX3CL1 | ICAM1 | LYZ | RB1 | FCGR3B |

| | | | | | |
|---|---|---|---|---|---|
| CCR2 | CX3CR1 | ICOS | MAGEA1 | RORC | GZMM |
| CCR4 | CXCL1 | ICOSLG | MAGEA1<br>2 | RUNX3 | HLA-DQA1 |
| CCR5 | CXCL10 | IDO1 | MAGEA4 | S100A12 | HLA-DRB1 |
| CD14 | CXCL11 | IFI27 | MAGEC2 | S100A8 | HLA-E |
| CD163 | CXCL12 | IFI35 | MELK | S100A9 | OAZ1 |
| CD19 | CXCL13 | IFI6 | MKI67 | SDHA | PF4 |
| CD1C | CXCL5 | IFIH1 | MLANA | SELL | PRR5 |
| CD2 | CXCL8 | IFIT1 | MLH1 | SH2D1A | STK11IP |
| CD209 | CXCL9 | IFIT2 | MMP9 | SIGLEC5 | TBC1D10B |
| CD244 | CXCR2 | IFIT3 | MRC1 | SLAMF7 | TPSAB1 |
| CD247 | CXCR3 | IFITM1 | MS4A1 | SNAI1 | UBB |
| CD27 | CXCR4 | IFITM2 | MS4A2 | SPIB | |
| CD274 | CXCR6 | IFNG | MS4A4A | STAT1 | |
| CD276 | CYBB | IL10 | MSH2 | STAT3 | |
| CD28 | DLL4 | IL10RA | MSH6 | STAT4 | |
| CD38 | EGFR | IL12RB2 | MTOR | TAP1 | |
| CD3D | EIF2AK2 | IL15 | MX1 | TBP | |
| CD3E | ENTPD1 | IL17A | MYC | TBX21 | |
| CD3G | EOMES | IL18 | NBN | TCL1A | |
| CD4 | FAS | IL1A | NCAM1 | TDO2 | |
| CD40 | FASLG | IL1B | NCR1 | TFRC | |
| CD40LG | FCAR | IL2 | NECTIN2 | TGFB1 | |

**Supplementary Table 3：** 289 immune-related genes, including housekeeping genes list.

**Supplementary Table 4: Cell type gene list**

| Cell type | Gene list |
| --- | --- |
| T cells | CD3D、CD3E、CD3G、CD6、SH2D1A、TRAT1 |
| B cells | BLK、CD19、FCRL2、MS4A1、PNOC、SPIB、TCL1A、TNFRSF17 |
| Mast cells | CPA3、HDC、MS4A2 |
| DC | CCL13、CD209、HSD11B1 |
| Macrophages | CD163、CD68、CD84、MS4A4A |
| Neutrophils | CEACAM3、CSF3R、FCAR、FPR1、S100A12、SIGLEC5 |
| Cytotoxic cells | CTSW、GNLY、GZMA/B/H、KLRB1、KLRD1、KLRK1、NKG7、PRF1 |
| Exhausted CD8 | PTGER4、LAG3、EOMES、CD244 |
| NK CD56 cell | KIR3DL1/2/3、IL21R |
| CD8 T cell | CD8B、CD8A |
| CD45 cell | PTPRC |
| Th1 cell | TBX21 |
| NK cell | NCR1 |
| Treg cell | FOXP3 |
| Terminally exhausted T cells | PDCD1、CTLA4、HAVCR2、ENTPD1、 ITGAE |
| Progenitor exhausted T cells | IL7R、GZMK、T-bet (TBX21) |

**Supplementary Table 4:** The genes list of 14 immune cell types in our study.

## Supplementary Table 5: Signatures/Scores gene list and respective references

| **Signatures/Scores** | **Gene/cell list** | References |
|---|---|---|
| T cell score | CD2, CD3D, CD3E, HLA-E, IL2RG, NKG7 | 1 |
| B cell score | CXCL13, CXCR6, IL18, IL2RG, LCK, PSMB10, TNFRSF4, TNFRSF14 | 2 |
| Immune signature | CD2, CD247, CD3E, GZMH, GZMK, NKG7, PRF1 | 3 |
| CTL score | CD8A, CD8B, GZMA, GZMB, PRF1 | 4 |
| CYT score | PRF1, GZMA | 5 |
| Chemokines score | CCL5, CCR5, CXCL9, CXCR6 | 6 |
| Angiogenesis score | VEGFA, PECAM1 | 7 |
| IFN-γ signature | CXCL10, CXCL9, HLA-DRA, IDO1, IFNG, STAT1 | 1 |
| T cell inflamed GEP score | CCL5, CD27, CD274, CD276, CD8A, CMKLR1, CXCL9, CXCR6, HLA-DQA1, HLA-DRB1, HLA-E, IDO1, LAG3, NKG7, PDCD1, PSMB10, STAT1, TIGIT | 1 |
| Total TILs score | B-cells, CD8 T cells, Cytotoxic cells, Exhausted CD8, Macrophages, NK CD56dim cells, T-cells | 8 |

**References:**


1. Ayers, M. et al. IFN-γ-related mRNA profile predicts clinical response to PD-1 blockade. J Clin Invest 127, 2930–2940 (2017).

2. Lundberg, A., Li, B. & Li, R. B cell-related gene signature and cancer immunotherapy response. Br J Cancer 126, 899–906 (2022).

3. Davoli, T., Uno, H., Wooten, E. C. & Elledge, S. J. Tumor aneuploidy correlates with markers of immune evasion and with reduced response to immunotherapy. Science 355, eaaf8399 (2017).

4. Jiang, P. et al. Signatures of T cell dysfunction and exclusion predict cancer immunotherapy response. Nat Med 24, 1550–1558 (2018).

5. Rooney, M. S., Shukla, S. A., Wu, C. J., Getz, G. & Hacohen, N. Molecular and genetic properties of tumors associated with local immune cytolytic activity. Cell 160, 48–61 (2015).

6. Miu, J. et al. Chemokine gene expression during fatal murine cerebral malaria and protection due to CXCR3 deficiency. J Immunol 180, 1217–1230 (2008).

7. Landt, S. et al. The utility of an in vitro angiogenesis score for prognosis assessment in patients with cervical cancer. Anticancer Res 31, 2645–2649 (2011).

8. Danaher, P. et al. Gene expression markers of Tumor Infiltrating Leukocytes. J Immunother Cancer 5, 18 (2017).

Study Title: Clinical research on PD-1 inhibitor plus Gemcitabine based chemotherapy as the treatment in advanced biliary tract cancer

Primary sponsor：Eastern Hepatobiliary Surgery Hospital Second,

Military Medical University

Study leader：Zhen-gang Yuan

Protocol No.:
Protocol Version: V 1.0
Date: Sep. 5, 2019

# 1. BACKGROUND

## 1.1 Disease Background

Biliary tract cancer (BTC) include Intrahepatic cholangiocarcinoma (ICC), Extrahepatic bile duct carcinoma (EBDC) and gallbladder cancers (GBC), R0 resection is the most efficient treatment option for BTC. However, patients initially presented with advanced disease due to the insidious onset of the disease and the lack of effective antitumor drugs result in a very poor prognosis that the 5-year survival rate is less than 5% [1]. 5-FU-based chemotherapy regimens have been the standard treatment for biliary tract tumors, with response rates ranging from 25 to 32% [2-3]. The addition of other drugs, such as cisplatin, doxorubicin, epirubicin, and methotrexate, was stopped in phase II clinical trials due to poor efficacy [4-6]. Until the ABC01 study in 2009 and the ABC02 study in 2010, gemcitabine combined with Gencitabine/cisplatin (GP) was established as the standard first-line therapy for BTC [7-8], The disease control rate (CR+PR+SD) was 75% in the phase II study and 81.4% in the phase III study. The median Time to progression (TTP) reached 8 months in the phase II clinical study, and the median Overall survival (OS) reached 11.7 months in the phase III clinical study. The risk of death was 36% lower than in the control group (gemcitabine alone).

Thereafter, ASCO GI published a phase III clinical study (JCOG1113, FUGA-BT) in Japan in 2018, which showed that gemcitabine combined with S-1 in advanced cholangiocarcinoma was non-inferior to GP with good tolerability [9]. Based on this, GS regimen, which is convenient to use and does not require hydration, could be considered as the standard treatment for advanced cholangiocarcinoma. A multicenter, single-arm, two-phase, phase II clinical study of a new treatment for advanced cholangiocarcinoma was published in JAMA Oncology in 2018, which revealed the efficacy and safety of a weekly Abraxane (Nab-P) combined with gemcitabine in patients with advanced unresectable and metastatic cholangiocarcinoma[10], The median PFS and OS were 7.7 months (95%CI, 5.4-13.1) and 12.4 months (95%CI, 9.2-15.9), respectively, and the ORR was 30%, which was significantly higher than that of gemcitabine plus oxaliplatin (20%) and gemcitabine plus cisplatin (19%). It can be considered that Nab-P combined with gemcitabine is an effective and safe alternative for advanced CCA. Recently, a study published in JAMA Oncology evaluated the promising efficacy of GP combined with Nab-P in patients with advanced BTC [11]. The results showed that Nab-P combined with GP prolonged mPFS and overall OS compared with GP. The median PFS was 11.8 (95% CI, 6.0-15.6) months and the mOS was 19.2 months (95% CI, 13.2 months to not reached). The partial response rate was 45% and the disease control rate was 84%.

Since the discovery of immune checkpoint inhibitors in the 1990s, the treatment of melanoma, kidney cancer, lung cancer and other tumors has changed dramatically. The NCCN Clinical Practice guideline (2019.v3) recommends Pembrolizumab for MSI-H/dMMR unresectable or metastatic hepatobiliary tumors. At the same time, more and more clinical studies of anti-PD-1 inhibitors applied in hepatobiliary tumors have been carried out. Multiple studies have found that the combination of anti-PD-1 inhibitors and chemotherapy significantly improves the efficacy, with significantly prolonged PFS and OS. On March 29th of this year, the National Medical Products Administration of China formally approved Merck's anti-PD-1 inhibitor pembrolizumab in combination with pemetrexed and platinum-based chemotherapy as the first-line therapy for patients with metastatic non-squamous non-small cell lung cancer (NSCLC) with epidermal growth factor receptor (EGFR) -negative and anaplastic lymphoma kinase (ALK) -negative. The anti- PD-1 inhibitor (Sintilimab, Daboshu) is a recombinant fully human monoclonal antibody against programmed death receptor 1, which is independently developed by Innovent Biologics (Suzhou) Co. Ltd.

Similar to other anti-PD-1 inhibitors, Sintilimab block the PD-1/PD-L1 pathway to enhance T-cell responses independent of PD-L1 expression and mediates PD-1 endocytosis while reducing its expression on membrane surface. Clinical studies have been carried out in lymphoma, gastric cancer, soft tissue sarcoma, nasopharyngeal carcinoma, esophageal squamous cell carcinoma and melanoma, with remarkable results and controllable safety. Clinical researches of Sintilimab have been carried out in lymphoma, gastric cancer, soft tissue sarcoma, nasopharyngeal carcinoma, esophageal squamous cell carcinoma and melanoma, with remarkable results and controllable safety.

In conclusion, we believe that the regimen of domestic anti-PD-1 inhibitor (Sintilimab, Daboshu) combined with gemcitabine-based chemotherapy is expected to improve the efficacy as the first-line therapy in patients with advanced BTC. In order to evaluate the efficacy and safety of this combined regimen, , we plan to begin a clinical study to observe the profiles of enrolled patients with advanced BTC.

## 1.2 Mechanism of Action of Sintilimab

Sintilimab (R&D code: IBI308) is a recombinant fully human IgG4 PD-1 monoclonal antibody. Multiple nonclinical trials *in vitro* have shown the blockade function of sintilimab of the PD-1 pathway, and completed nonclinical studies on pharmacodynamics, animal PK and toxicology indicating that sintilimab was characterized by clear targets, reliable source of cell lines, and good stability, and demonstrating good activity in all completed nonclinical studies. Refer to "Investigator's Brochure" for detailed study results. By February 28, 2018, a total of 5 studies and 371 cancer patients were treated with sintilimab, and the overall safety profile was similar to anti-PD-1 monoclonal antibodies approved abroad (Jianming Xu, et al. 2018CSCO).

## 1.3 Early Clinical Data of Sintilimab in Solid Tumors

The phase Ia dose-escalation trial of sintilimab was initiated in Sep. 2016 (study code: CIBI308A101-1a). Subjects with advanced solid tumors who had failed standard treatment were enrolled in phase Ia and the dose escalation decision followed the standard "3 + 3" design to evaluate 4 dose levels (1 mg/kg, 3 mg/kg, 200 mg, and 10 mg/kg) of sintilimab. After the completion of 1 mg/kg dose administration, subjects were randomized at a 1:1 ratio to either 3 mg/kg or 200 mg dose group for independent evaluations. Dose-limiting toxicity (DLT) was observed for 28 days after the first dose for each dose group. After completion of DLT observation, subjects were treated with sintilimab Q2W (1 mg/kg, 3 mg/kg, or 10 mg/kg) or Q3W (200 mg) until progressive disease (PD), intolerable toxicity, withdrawal of informed consent form (ICF), or other reasons requiring treatment discontinuation (whichever occurred first).

PK studies of patients with advanced solid tumors in phase Ia showed that the serum drug concentration of sintilimab (administered by intravenous infusion) gradually increased since the start of the infusion, reached Cmax after the infusion, and then slowly decreased. Within the dose range of 1–10 mg/kg, the sintilimab exposure in vivo had a dose-proportional increase, suggesting linear kinetic characteristics. In subjects with solid tumors, after the single dose of sintilimab, the elimination half-life (Geo.Mean [CV%]) was 14.4 [28.9%] days, the clearance rate was 11.5 [42.5%] mL/h, the steady-state distribution volume was 5.43 [34.4%] L, and the apparent distribution volume was 5.77 [33.2%] L, showing comparable PK characteristics to similar marketed anti-PD-1 antibodies (nivolumab and pembrolizumab).

In the phase Ia pharmacodynamics study of patients with advanced solid tumors, a single dose of sintilimab at 1 mg/kg (N = 3) could achieve rapid (24 h) saturation (mean ≥ 95%) of the PD-1 receptors on

the surface of peripheral CD3+ T cells of solid tumor subjects and could maintain the occupancy level during the study period (28 days) of continuously reducing drug concentrations and in therapy of multiple doses that were given consecutively. The dose groups of 3 mg/kg (N = 3), 200 mg (N = 3), and 10 mg/kg (N = 3) showed comparable results of PD-1 occupancy to the 1 mg/kg, suggesting no dose or concentration dependence within the range of 1–10 mg/kg for PD-1 receptor occupancy.

Sintilimab also showed good anti-tumor activity in subjects with multiple advanced solid tumors who had failed standard treatment. The evaluation as per RECIST V1.1 showed the best overall efficacy that 2 subjects achieved PR and 2 achieved SD. As of Dec. 9, 2017, neither dose-limiting toxicity (DLT) nor unexpected AE had been observed in the completed phase Ia study.

The CIBI308A101-Ib study was a multi-center phase Ib clinical study in Chinese patients with advanced solid tumors. Cohort C mainly included patients with locally advanced, relapsed, or metastatic NSCLC that failed the second-line or above standard treatments, who were treated with sintilimab monotherapy at 200 mg Q3W. As of Sep. 1, 2018, a total of 37 patients were enrolled in this cohort including 34 evaluable patients. All patients received at least one administration of sintilimab, with a median of 6 (1–18) administration cycles. The evaluation based on RECIST1.1 showed that the objective response rate (ORR) was 14.7% and the disease control rate (DCR) was 52.9%, with partial response (PR) observed in 5 patients (14.7%) and stable disease (SD) observed in 13 patients (38.2%);

CIBI308A101-Ib Cohort D mainly included patients with advanced, relapsed, or metastatic non-squamous NSCLC who had not received prior systematic anticancer therapy, and the patients were treated with sintilimab in combination with pemetrexed and cisplatin. As of Sep. 1, 2018,

a total of 21 subjects were enrolled and administered with at least one dose of the study drug, with a median administration cycle of 10 (1–19). A total of 19 subjects received at least one imaging assessment. The evaluation of existing data based on RECIST 1.1 showed that the ORR of patients with a best overall response (BOR) was 68.4%, with PR in 13 subjects, SD in 3 subjects, and PD in 3 subjects.

CIBI308A101 phase Ib cohort E mainly included patients with advanced, relapsed, or metastatic squamous NSCLC who had not received prior systematic anticancer therapy. The patients were treated with sintilimab in combination with gemcitabine and cisplatin. As of Sep. 1, 2018, a total of 20 patients had been enrolled. All patients received at least one dose of sintilimab and 17 patients completed at least one efficacy evaluation, of which 11 (64.7%) achieved PR, and 6 (25.3%) achieved SD; the ORR was 64.7%, the DCR was 100%, and the treatment-emergent adverse event (TEAE) ≥ grade 3 was 5.0%, indicating good efficacy and safety.

In a multi-center, single-arm, phase II clinical study (ORIENT-1) on sintilimab in relapsed or refractory classic Hodgkin lymphoma, a total of 96 patients were enrolled. The study results showed that sintilimab monotherapy had definite efficacy and good safety in this group of patients. As of Apr. 16, 2018, the IRRC-assessed ORR for the full analysis set (N = 92) was 80.4%, CR was 33.7%, and DCR was 97.9%. The median duration of response was not reached, and at the time of analysis, 62 out of 74 patients (84.8%) with CR and PR were still in response. At least 1 treatment-related adverse event occurred in 89 (92.7%) patients, most of which were grade 1–2. The most common treatment-related adverse event was fever (39/96, 40.6%), which mostly occurred during the first injection and recovered within 1 day. The most common immune-related adverse event was hypothyroidism (19/96, 19.8%) and all were grade 1–2; 3 patients (3.1%) discontinued treatment due to adverse events; no patient died.

A number of clinical studies of sintilimab for the treatment of various tumors were conducted subsequently. As of Feb. 28, 2018, a total of 5 studies were conducted with 371 tumor patients who received sintilimab.

The overall safety profile was similar to those of anti-PD-1 monoclonal antibodies approved overseas.

The dose of sintilimab in this study was determined as 200 mg Q3W, based on preliminary pharmacokinetic and pharmacodynamic results, acceptable safety events, and potential individual differences.

### 1.4 Risk/Benefit and Ethical Assessments of Sintilimab

Given the mechanism of action underlying IBI308 and the clinical safety information on products with similar mechanisms, the main AEs during this clinical trial will possibly be the immune-mediated inflammatory resulted from the activation of immune system, e.g. pneumonitis, colitis, hepatitis, renal insufficiency, and endocrine events. According to the available clinical data, anti-PD-1 monoclonal antibodies are well-tolerated despite of high incidence of adverse reactions. Treatment termination due to adverse reactions only occurs in a small number of patients and most events resolve after appropriate interventions. Early symptoms of immune-related adverse events (irAEs) vary. Therefore, the investigator must closely monitor early signs and symptoms of irAEs during the trial, make correct judgments timely, adjust the dose according to S the protocol, and provide effective treatment measures to reduce the subject risks. Meanwhile, subjects with autoimmune diseases should be excluded from the trial to avoid the original disease aggravation due to the activation of immune system.

## 2. STUDY OBJECTIVES

### 2.1 Primary Objective

To evaluate the efficacy of sintilimab combined with Gemcitabine -based chemotherapy as first-line therapy in patients with advanced biliary tract cancer.

### 2.2 Secondary Objective

To evaluate the safety and feasibility of sintilimab combined with Gemcitabine -based chemotherapy as first-line therapy in patients with advanced biliary tract cancer.

### 2.3 Exploratory Study Objectives

To explore the biomarkers related to the efficacy of sintilimab combined with Gemcitabine -based chemotherapy as first-line therapy in patients with advanced biliary tract cancer.

## 3 STUDY ENDPOINT

### 3.1 Primary Endpoint

Overall survival (OS).

### 3.2 Secondary Endpoints

Objective response rate（ORR）、progression-free survival（PFS）、Disease control rate（DCR）、Duration of response (DOR)、safety.

### 3.3 Exploratory Endpoint

Correlation between biomarkers and therapeutic response to therapy.

## 4 STUDY POPULATION

This was a single-arm study, and no randomization was used. To determine the sample size for this clinical trial, overall survival (OS) with standard of care chemotherapy (Gem/Cis) as the historical control was assumed to be 9.5 months based on previously reported data and the notable features of the high proportion of ICC patients in our center. The addition of sintilimab to chemotherapy would expect to improve the OS to 16.0 months. Given an accrual period of 24 months, a maximum follow-up time of 48 months, at the significance level of 0.05, to achieve the power of 0.8, the number of events required is 23. Equivalently, a sample size of 30 is needed. The sample size result is based on a one-sided test with exponential assumption for survival time. 30 treatment-naive Patients with advanced biliary tract cancer will be enrolled in this study.

### 4.1 Inclusion Criteria

1. Histologically, cytologically confirmed biliary tract cancer;
2. with at least one measurable lesion as the target lesion according to the Response Evaluation Criteria in Solid Tumors (RECIST) criteria (V.1.1);
3. Patients aged 18 to 75;
4. An Eastern Cooperative Oncology Group performance status of 0–2;
5. Adequate haematological function (white blood cell count > 3.0 x10^9/L, platelet count > 100 x10^9/L);
6. Adequate liver function (bilirubin ≤ 1.5 times the upper limit of normal (ULN);
7. Adequate renal function (creatinine clearance > 60 mL /min; creatinine < 120μmol /L);
8. Had no heart failure, no uncontrollable chest pain, and no myocardial infarction within 12 months prior to the study;
9. Expected survival≥3 months;
10. Signed an informed consent form.

### 4.2 Exclusion Criteria

1. Received systemic treatment previously, including chemotherapy, targeted and immunotherapy; 2. Associated secondary malignancies or other type of tumors with metastasis to the brain or meninges within 3 years prior to study initiation;
3. Drug contraindications: severe hypertension, high risk of bleeding, severe nephrotic syndrome, etc.;
4. With chronic diarrhea or colorectal inflammatory conditions, or untreated obstruction or incomplete obstruction affecting systemic administration;
5. Active infection or other severe infection that may prevent the patient from receiving planned management (bacterial cholangitis, which destroys a branch of the biliary tract over a long period of time without control);
6. Cardiac insufficiency, unstable angina pectoris, congestive heart failure, myocardial infarction occurred within 6 months before enrollment, serious uncontrollable arrhythmia; 6. Participated in other clinical trials;

7. Have a history of uncontrollable substance abuse or mental disorder;
8. Patients with concomitant diseases that, in the investigator's judgment, may seriously endanger their own safety or may interfere with the completion of the study;
9. Participated in other clinical trials;
10. Pregnant or lactating women;
11.Individuals under corrective monitoring or supervision.

# 5 STUDY DESIGN AND PLAN

## 5.1 Discussion of Study Design

This is a single-arm clinical study aimed to evaluate the clinical efficacy and safety of sintilimab combined with Gemcitabine-based chemotherapy as as first-line therapy in patients with advanced biliary tract cancer. Treatment-naive Patients who pathologically confirmed biliary tract cancer will be enrolled in this study after sign the ICF.

## 5.2 Drugs and Treatments Administered

### 5.2.1 Drugs

**Anti-PD-1 inhibitor**（Sintilimab）**：** Sintilimab is administered by intravenous infusion at a recommended dose of 200 mg once every three weeks for up to 2 years or until disease progression, intolerable toxicity,.
**chemotherapy：** Gemcitabine-based chemotherapy is administered every three weeks. The combination regimen was continued until disease progression, intolerable toxicity, or completed 6-8 cycles treatment.

### 5.2.2 Adverse Drug Reactions and Dose adjustment

**Anti-PD-1 inhibitor：** Sintilimab dose adjustment is not permitted throughout the study, and the principles for dose interruption and permanent discontinuation of sintilimab are shown in the table below.

| **Sintilimab-related AEs** | **Severity** | **Dose adjustment** |
|---|---|---|
| Pneumonia | Grade 2 pneumonia | Dose interruption[a] |
| | Recurrent grade 2 pneumonia, grade 3 or 4 pneumonia | Permanent discontinuation |
| Diarrhea/colitis | Grade 2 or 3 diarrhea or colitis | Dose interruption[a] |
| | Grade 4 diarrhea or colitis | Permanent discontinuation |
| Hepatitis | Grade 2 AST, ALT, or TBIL elevation for subjects with normal AST, ALT, or TBIL at baseline; AST, ALT, or TBIL elevation by ≥ 50% (meeting the criteria for grade 2) lasting for < 7 days for subjects with AST, ALT, or TBIL > ULN at baseline | Dose interruption[a] |
| | Grade 3 or 4 AST, ALT, or TBIL elevation for subjects with normal AST, ALT or TBIL at baseline; AST, ALT, or TBIL elevation of ≥ 50% (reaching the requirements of grade 3 or | Permanent discontinuation |

| Sintilimab-related AEs | Severity | Dose adjustment |
|---|---|---|
| | 4) for ≥ 7 days for subjects with AST, ALT, or TBIL > ULN at baseline | |
| Skin adverse reactions | Grade 3 | Dose interruption[a] |
| | Grade 4 Stevens-Johnson syndrome (SJS) or toxic epidermal necrolysis (TEN) | Permanent discontinuation |
| Hypophysitis | Grade 2 or 3 hypophysitis | Dose interruption[b] |
| | Grade 4 hypophysitis | Permanent discontinuation |
| Adrenocortical insufficiency | Grade 2 adrenocortical insufficiency | Dose interruption[b] |
| | Grade 3 or 4 adrenocortical insufficiency | Permanent discontinuation |
| Thyroid function abnormal | Symptomatic grade 2 or 3 hypothyroidism, or grade 2 or 3 hyperthyroidism | Dose interruption[b] Permanent discontinuation |
| | Grade 4 hypothyroidism, or grade 4 hyperthyroidism | Permanent discontinuation |
| Diabetes/hyperglycemia | Grade 3 hyperglycemia | Dose interruption[b] |
| | Grade 4 hyperglycemia | Permanent discontinuation |
| Nephritis | Grade 2 or 3 increased blood creatinine | Dose interruption[a] |
| | Grade 4 increased blood creatinine | Permanent discontinuation |
| Thrombocytopenia | Grade 3 thrombocytopenia | Dose interruption[a] |
| | Grade 4 thrombocytopenia | Permanent discontinuation |
| Other AEs | Grade 3 or 4 hyperamylasemia or lipase increased<br>Grade 2 or 3 pancreatitis<br>Grade 2 myocarditis*<br>Grade 2 or 3 other AEs (first occurrence) | Dose interruption[a] |
| | Grade 4 pancreatitis or relapsed pancreatitis of all grades<br>Grade 3 or 4 myocarditis<br>Grade 3 or 4 encephalitis<br>Grade 4 other AEs (first occurrence) | Permanent discontinuation [c] |

a: Resume administration of the study drug after symptoms improve to grade 0–1 or baseline levels.

* The safety of resuming treatment with sintilimab after myocarditis returns to Grade 0–1 in severity is yet unclear

b: Resume the administration if hypophysitis, adrenocortical insufficiency, thyroid function insufficiency/hypothyroidism, or type I diabetes mellitus is adequately controlled and only physiological hormone replacement therapy is required.

c: For grade 4 laboratory abnormalities, whether to terminate the treatment shall be determined based on clinical signs/symptoms and the clinical judgment of the investigator.

Permanently discontinue sintilimab if treatment-related adverse reactions do not return to Grade 0–1 or the baseline level within 12 weeks after the last dose, except:

1) If corticosteroids are used for the treatment of immune-related adverse reactions, the maximum discontinuation of sintilimab due to corticosteroid reduction should not exceed 12 weeks. In these cases, a comprehensive investigator evaluation is required to determine whether sintilimab can be continued. Imaging tests to efficacy assessment were performed as planned and were not affected by the drug suspension.

2) Sintilimab was suspended for more than 12 weeks due to treatment of AE unrelated to itself. In these cases, a comprehensive investigator evaluation is required to determine whether sintilimab can be continued. Imaging tests to efficacy assessment were performed as planned and were not affected by the drug suspension.

## 5.3 The principle of Sintilimab Resume Administration

Resume administration of Sintilimab after symptoms improve to grade 0–1 or baseline levels， and ECOG PS 0-1.

## 5.4 Management of sintilimab infusion-related reactions

Sintilimab may lead to severe or life-threatening infusion-related reactions, including severe hypersensitivity or allergic reactions. Signs and symptoms usually occur during or after the drug infusion and usually resolve within 24 h after the infusion completion. The guidelines for management of sintilimab infusion-related reactions are shown in the table below.

**Table 3. Guidelines for management of sintilimab infusion-related reactions**

| NCI CTCAE Grades | Treatment | Premedications for subsequent infusions |
|---|---|---|
| Grade 1<br>Mild reaction; infusion interruption not indicated; intervention not indicated | According to patient's medical indications, monitor the vital signs closely until the subject is stable as determined by the investigator. | None |
| Grade 2<br>Treatment or infusion interruption required, but responds promptly to timely symptomatic treatment (e.g. antihistamines, non-steroidal anti-inflammatory drugs [NSAIDS], anesthetics, intravenous fluids replacement); prophylactic medications indicated for ≤ 24 h | Stop the infusion and monitor symptoms.<br>Other appropriate treatments include but are not limited to:<br>IV infusion<br>Antihistamines<br>NSAIDS<br>Acetaminophen anesthetics<br>According to patient's medical indications, monitor the vital signs closely until the subject is stable as determined by the investigator. | The following premedications are recommended within 1.5 h (± 30 min) prior to sintilimab infusion:<br><br>Diphenhydramine 50 mg PO (or equivalent antihistamines).<br><br>Acetaminophen |

| NCI CTCAE Grades | Treatment | Premedications for subsequent infusions |
|---|---|---|
| | If symptoms resolve within 1 h after the interruption of the infusion, then the infusion can be resumed at 50% of the original infusion rate (e.g. from 100 mL/h to 50 mL/h). Otherwise, interrupt the treatment until symptoms resolve. Premedications should be given for subsequent infusions.<br>If grade 2 toxicities occur despite of adequate premedications, the study drugs should be permanently discontinued. | 500–1000 mg PO (or equivalent antipyretics). |
| Grade 3 or 4<br>Grade 3:<br>Prolonged (i.e. not rapidly responsive to symptomatic medication and/or brief interruption of infusion); recurrence of symptoms following initial improvement; hospitalization indicated for other clinical sequelae (e.g. renal impairment, pulmonary infiltration)<br>Grade 4:<br>Life threatening; pressors or ventilatory support indicated | Discontinue the infusion.<br>Other appropriate treatments include but are not limited to:<br>Epinephrine**<br>IV infusion<br>Antihistamines<br>NSAIDS<br>Acetaminophen anesthetics<br>Oxygen<br>Pressors<br>Corticosteroids<br>According to patient's medical indications, monitor the vital signs closely until the subject is stable as determined by the investigator.<br>Hospitalization may be indicated.<br>**Epinephrine should be used immediately for allergic reactions.<br>The study drugs should be permanently discontinued. | Not applicable |
| Appropriate first-aid equipment should be provided in the ward and physicians should be available at all times during the administration.<br>For more information, refer to "Common Terminology Criteria for Adverse Events" (CTCAE) V5.0 (http://ctep.cancer.gov) | | |

# 6 EFFICACY EVALUATION AND SAFETY EVALUATION

## 6.1 Efficacy Evaluation

RECISTv1.1(see appendix 1) will be mainly used for this study, and tumor response will be assessed every 6 weeks. Meanwhile, subject will follow up with accepted examination of blood routine, liver and kidney functional， electrolytes, coagulation function, tumor indicators, immune-related indicators, etc.

## 6.2 Safety Evaluation

The following safety and tolerance of treatment will be evaluated in this study

- ➢ AE： in this study， AEs will be reported by the subject (or a caregiver, surrogate, or legal representative of the subject).
- ➢ ECOG PS score（see appendix 2）
- ➢ Clinical laboratory tests
- ➢ Vital signs (pulse, temperature, blood pressure, respiratory rate)
- ➢ Physical examination

Any clinically significant abnormalities that persist at the end of the study will be closely followed until the problem is resolved or the patient reaches a clinically stable endpoint.

Safety analysis is the analysis of the incidence of AEs. AEs will be reported from the time subjects signed the ICF until 30 days after the last dose, and AEs were assessed at each follow-up cycle according to the National Cancer Institute Common Naming Standard for Adverse Events (NCI CTCAE) version 5.0.

## 6.3 Monitoring Indicators

（1）Patient's vital signs: temperature, pulse, respiration, blood pressure

（2）Blood routine, urine routine, fecal routine (including at least the beginning and end of each cycle)

（3）Liver and kidney function, electrolytes, coagulation function, amylase, blood lipids, creatine kinase, thyroid function, antinuclear antibodies, lymphocyte subsets analysis, tumor indicators (at least including AFP, PVIKA, CEA, CA199)

（4）12-lead electrocardiogram (ECG) and X-ray sternum

（5）Liver enhanced CT or MRI(consistent with baseline)

（6）CT of head and lung (if necessary)

（7）bone scanning (if necessary)

（8）NGS gene testing

# 7 STUDY PROCEDURES

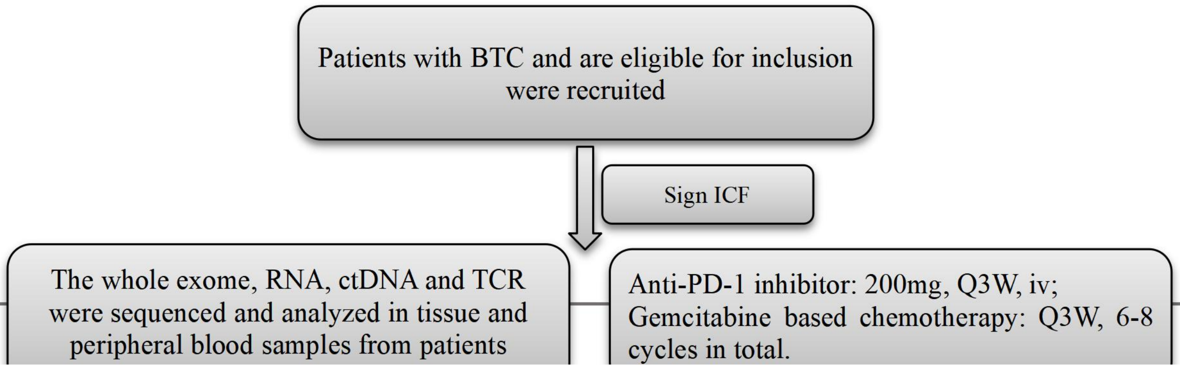

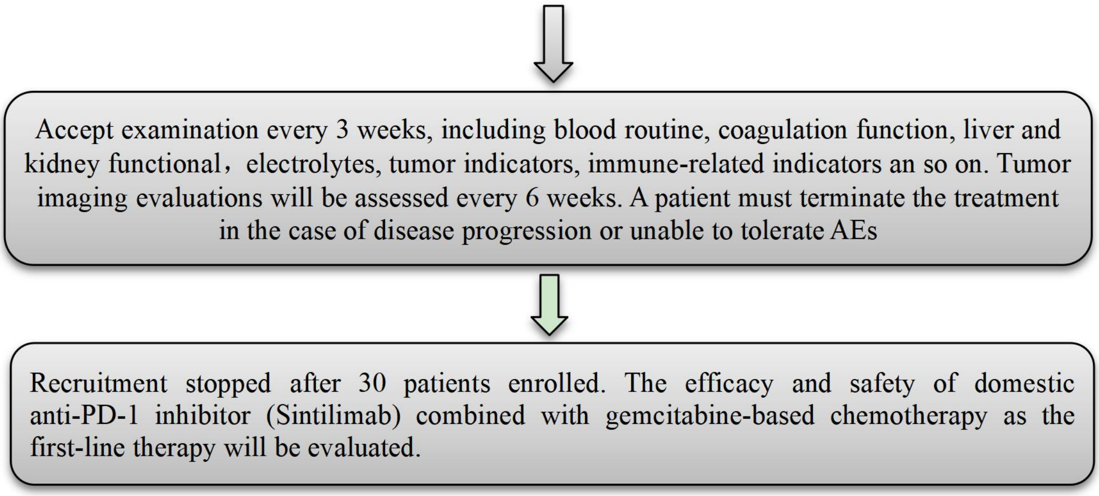


# 8 DRUG MANAGEMENT

## 8.1 Management of Sintilimab

The study drug of this study is sintilimab (IBI308). Sintilimab must be stored at 2–8 °C, away from light, and avoid freezing. All experimental drugs are transported to each site in cold chain, and should be kept and distributed by special personnel.

The investigational drugs should be stored in a refrigerator only accessible to the authorized personnel. After receiving the study drugs, the investigator should ensure that the temperature during transport is maintained within the specified range, sign for receipt upon verification, and store the drugs at the specified temperature. If abnormalities of the storage temperature during either the transportation or storage at the study site arise, the drugs should be moved to an environment in the specified temperature as soon as possible and should not be administered to the subjects at the moment. Innovent should be timely notified and the advice of Innovent should be followed.

All the study drugs provided by the sponsor should only be used for this clinical trial. Any usage of the study drug other than those specified in the protocol are prohibited. The investigator must agree not to provide the investigational drugs to anyone unrelated to this trial.

## 8.2 Drug Return and Destruction

The used containers for sintilimab injections can be destroyed on-site according to the appropriate guidelines and operating procedures established by study sites and local authorities. Upon the completion or discontinuation of the study, all unused or expired study drugs must be returned for destruction.

## 8.3 Study Drug-related Records

The designated personnel of the Site shall make timely records of the receipt, distribution, use, inventory, destruction, recovery and destruction of the drugs in accordance with the requirements of relevant regulations and guidelines.

# 9 ADVERSE EVENT REPORTING

## 9.1 Definitions of Adverse Event

An adverse event (AE) is defined as any adverse unexpected medical event within the period from the signing of the informed consent form, regardless of whether or not considered as related to the study drug. AEs include but are not limited to the following:

- Worsening of pre-existing (before enrollment) medical conditions/diseases (including symptoms, signs, and laboratory test abnormalities);
- Any new adverse medical conditions (including symptoms, signs, and newly diagnosed diseases);
- Clinically significant laboratory test abnormalities.

AEs include SAEs and non-SAEs.

## 9.2 Definitions of Serious Adverse Event

According to ICH and EU pharmacovigilance guidelines for medical products for human use,

a serious adverse event (SAE) is an unexpected medical event that occurs at any drug dose and meets any one of the following criteria:

Leading to death;

Life-threatening (the subject is under threat of death at the time of the event, excluding events that may theoretically lead to death if the situation becomes more severe);

Leading to hospitalization or prolonged hospitalization, excluding the following cases:

Rehabilitation facility

sanatorium

Routine emergency room admission

Same-day surgery (e.g. outpatient/same-day/ambulatory surgery)

Hospitalization or prolonged length of stay that is not associated with SAE. For example, there were no new adverse events or exacerbations of pre-existing conditions (e.g., to check for laboratory abnormalities that persisted prior to the trial); Hospital admissions for management reasons (e.g., annual routine medical examinations); Hospitalization during the clinical trial as specified by the protocol (e.g., operation as required by the protocol); Elective hospitalizations not associated with worsening AE (e.g., elective surgery); Scheduled treatments or surgical procedures should be documented throughout the protocol and/or in the baseline data of the individual subject; Hospital admission solely for blood use.

Leading to a permanent or significant disability/function loss;

Causing deformities/birth defect;

Being suspected to transmit any source of infection through the study product;

Significant medical events*.

*The decision to adopt rapid reporting process in situations other than those listed above shall be made based on medical and scientific judgment. For example, significant medical events may not be immediately life-threatening or lead to death or hospitalization, but may harm the patient or may require therapeutic intervention to prevent occurrence of the above situations. They are usually considered SAEs.

### 9.3 Assessment of Adverse Events

The investigator will evaluate all AEs according to the NCI "Common Terminology Criteria for Adverse Events" (CTCAE) V5.0. AEs with altered CTCAE grade will be documented in the AE case report form (CRF)/worksheet. All AEs, regardless of the CTCAE grade, must be assessed for whether they are SAEs or not.

### 9.4 AE Documentation

The investigator should document AEs and SAEs using medical terms and concepts. Colloquialisms/abbreviations should be avoided. All AEs (including SAEs) shall be documented on the AE forms in the CRFs.

### 9.5 Adverse Event Collection and Times

The investigator should learn about AEs by asking the subjects non-leading questions.

After signing the informed consent form but before starting the study treatment, only the SAEs caused by the interventions procedures specified in the study protocol (for example, invasive procedures such as biopsy) should be reported.

All AEs, including SAEs, that occur from the initiation of the treatment with the study drug to 30 days after the last dose or patient starts a new treatment （Whichever comes first）shall be collected, regardless of whether they are related to the study treatment and whether they are observed by the investigator or self-reported by the subject. Thereafter, the investigator shall report any SAEs that are considered related to the study drugs or procedures in 30 days after the last dose.

### 9.6 Follow-up of AEs

The AE should be followed until the events return to the baseline values or grade 0–1, or until the investigator believes that no further follow-up is required for reasonable reasons (if the event cannot be resolved or has already been improved). If the event cannot be resolved, a reasonable explanation should be documented in the CRF. The outcome of an AE/SAE and the date should be documented in the CRF and medical record, regardless of whether the event is related to the study drugs.

## 9.7 Contents of AE Documentation

The investigator should document the complete information of any AE, including diagnosis (in the absence of diagnosis, symptoms and signs including laboratory test abnormalities should be documented), time and date of occurrence (if applicable), CTCAE severity grade and alteration (for events ≥ grade 3), whether it is an SAE, measures taken for the study drugs, treatment for the AE, outcome of the event, and causality between the event and study drugs.

For an SAE, the investigator shall also provide the date when the AE meets the criteria for an SAE, the date when the investigator is informed of the SAE, the reason of being an SAE, date of hospitalization, date of discharge, possible cause of death, date of death, whether an autopsy has been performed, causality assessment of the study procedures, causality assessment of other drugs, and other possible causes of the SAE. The investigator shall provide the rationales of the causality and a description of the SAE. In the SAE description, the following shall also be included: number, age, gender, height, and weight of the subject; indication for and the stage when receiving the investigational drug, and overall condition; clinical disease course including occurrence, development, outcome, and result of the SAE; laboratory results related to the SAE (the time of the examination, units, and normal ranges must be provided); medical history, onset and duration of concurrent diseases related to the SAE; medication history and initiation, duration, and dosage of concomitant medications related to the SAE; initiation, duration, and dosage of the study drug.

Descriptions of the AE are as follows:

- Diagnosis, signs, and symptoms

The diagnosis, if there is one, should be documented in the CRF rather than individual signs and symptoms (e.g. hepatic failure rather than jaundice, transaminases increased, and flapping tremor). Signs and symptoms should be reported as separate AEs/SAEs if unable to be attributed to the diagnosis. If it is determined that the signs and symptoms are caused by the diagnosis, then only the diagnosis which includes the signs and symptoms shall be reported. The record of signs and symptoms shall then be deleted for AE. An updated follow-up report shall be submitted for in the case of SAE.

- AEs secondary to other events

Generally, AEs secondary to other events (such as result of another event or clinical sequelae) should be documented as the primary event, unless the event is severe or is an SAE. However, clinically significant secondary events should be recorded as independent AEs in the eCRFs if they occur at different time points from the primary event. If the relationship between events is unclear, document them as separate events in the CRFs.

- Persistent or recurrent AEs

A persistent AE refers to an event that does not resolve and is ongoing between two assessment time points. These AEs should only be documented once in the CRFs. The initial severity level should be documented, and the information should be updated if the event exacerbates to record the most severe level of the event.

Recurrent AEs refer to AEs that have resolved between the two time points of assessment but subsequently occur again. These events should be independently documented in the eCRFs.

- Laboratory test abnormalities

All clinically significant laboratory test abnormalities are reported as AEs. The investigators have responsibilities to review all the laboratory test abnormalities and determine whether the abnormalities should be reported as AEs.

- Death

During the entire course of the study, all deaths should be documented in the Mortality Report Form in the CRFs, regardless of the causal relationship with the study drug. If the investigator judges the death that occurs during the AE reporting period specified in the study protocol as separate disease progression, the death should only be recorded on the CRF page of study completion/early termination. All other deaths in the study, regardless of the relationship with the study drugs, must be recorded as AE and reported to relevant authorities.

- Pre-existing medical conditions

Symptoms/signs presenting during the screening period will be recorded and reported as AEs only if their severity level, frequency, or property becomes aggravated (except for worsening of the disease under study). The relative change from previous condition should be expressed, such as "increased frequency of headaches".

- Disease progression

Disease progression is defined as the worsening of subject condition, the appearance of new lesions, or the progression of the primary lesion, caused by the primary tumor that the investigational drug is targeting. Expected disease progression should not be reported as an AE. Any deaths, life threatening events, hospitalization or prolonged hospitalization, permanent or significant disability/incapacity, congenital anomaly/birth defects, or other important medical events resulted from symptoms and signs of the expected disease progression should not be reported as an SAE.

- New anti-tumor therapy

If the subject initiates new anti-tumor therapy within 30 days after the last dose, then only SAEs considered to be related to the study drugs are required to be documented and reported.

## 9.8 SAE and Pregnancy Rapid reporting

SAE report

The SAE reporting period is for serious adverse events occurring within 30 days (inclusive) from the signing of informed consent to the date of the last dose. All SAEs as determined by the investigator shall be recorded in a "Serious Adverse Event Report Form" within 24 h after the investigator is informed, and then reported to Innovent (drugsafety@innoventbio.com) and to the national regulatory authorities and ethics committees in accordance with Chinese regulatory requirements. SAEs that occurred outside of this period but are considered to be related to the study drug should also be reported.

Pregnancy

Safety risk of embryotoxicity exists in the similar kind of drugs. All subjects with childbearing potential participating in the clinical trial must take effective contraceptive measures.

During the study, if a female subject exposed to the study drugs becomes pregnant, the subject must be withdrawn from the study. The investigator must report to Innovent within 24 h of learning the pregnancy and fill in the "Innovent Clinical Study Pregnancy Report/Follow-Up Form". During the study, if the female partner of a male subject exposed to the study drugs becomes pregnant, the subject will continue the study. The investigator must report to Innovent within 24 h of learning the pregnancy and fill in the "Innovent Clinical Study Pregnancy Report/Follow-Up Form".

The investigator must continuously monitor and follow up on the outcome of the pregnancy until 8 weeks after the delivery. The outcome shall be reported to Innovent.

If the outcome of the pregnancy is stillbirth, spontaneous abortion, fetal malformation (any congenital anomaly/birth defects), or induced abortion for medical reasons, it should be considered as an SAE, and the event is required to be reported in accordance with SAE procedures and time limits.

If the subject also experiences an SAE during pregnancy, the event should be reported according to SAE procedures.

### 9.9 Immune-related AEs

Given that the mechanism of sintilimab is to induce T-cell activation and proliferation, it is possible that immune-related adverse events (irAE) were observed during this study. Subjects should be monitored for signs and symptoms of irAE. If there is no clear alternative cause (e.g., infection), the signs or symptoms of the disease occurring in the subject should be considered to be immune system related.

The guidelines for sintilimab dose adjustment and management of adverse events are described in Sections 6.12 and 6.13 of protocol.

## 10 QUALITY ASSURANCE AND QUALITY CONTROL

According to the GCP guidelines, it is the sponsor's responsibility to implement and maintain a quality assurance and quality control system in accordance with the corresponding standard operating procedures to ensure that the implementation of clinical trials as well as the data collection, recording, and reporting comply with the requirements of the protocol, GCP, and local laws and regulations.

## 11 ETHICS

### 11.1 Ethics Committee

The sponsor or designee will prepare the relevant documents including the trial protocol, ICF, Investigator's Brochure, subject recruitment materials or advertising, and other documents required by regulations, which are to be submitted to the corresponding Ethics Committee (EC) in of the study site for approval. The written approval from the EC must specify the name, number, version number of the study protocol and other documents (such as ICF), and date of approval. The investigator shall notify the sponsor of written comment on the delay, suspension, or reapprove from EC.

The study site must follow the requirements of the EC in the study site. This may include revisions on

protocol, ICF, and recruitment materials should be submitted to the EC for approval. Local safety reports should be made and updated regularly in accordance with the regulations from the EC, and the final report should be submitted. All the above documents and EC approvals must be provided to the sponsor or designee.

## 11.2 Ethics of this Study

The implementation of this study and the access to Informed consent form shall comply with the requirements of the ICH, GCP, and local laws and regulations.

The GCP is an international ethical and scientific specification for designing, conducting, recording and reporting clinical trials that involve the participation of human subjects. This study will be conducted in accordance with the GCP and relevant national regulations and in accordance with the relevant ethical principles of "the Declaration of Helsinki" to protect the rights, safety, and health of the subjects.

The investigator is required to follow the procedures specified in this protocol and must not change the procedures without the permission from the sponsor. Protocol deviations, if any, must be reported to the EC, sponsor, or regulatory authorities.

## 11.3 Subject Information and Informed Consent Form

Before the start of any study procedure, the informed consent form (ICF) is used to explain the risks and benefits of this study to potential participants. The language used on the informed consent form should be straightforward. It should be clarified in the ICF statement that the ICF is voluntarily signed, and the risks and benefits of participating in this study should be clearly outlined. It should also be pointed out that subjects are free to withdraw from the study at any time. The investigator may only enroll a subject after fully explaining the details of the study, answering questions to the subject's satisfaction, giving the subject sufficient time for consideration, and obtaining written consent from the subject or the subject's legal representative. All signed ICFs must be kept in the investigator files or in the subject folders.

The investigator is responsible for explaining the contents of the ICF and obtaining the ICF signed and dated by the subject or the subject's legal representative prior to the study. After that, the investigator should provide the subject with a copy of the signed ICF. The investigator must record the process of informed consent in the source documents of the trial.

## 11.4 Data Protection

An ICF shall include (or in some cases, use separate files together) information on data and privacy protection.

Take precautions to ensure the confidentiality of the documents and prevent the disclosure of information that can identify a subject. However, under special circumstances, some personnel may have access to the genetic data and individual identification number of a subject. For example, in the event of a medical emergency, the sponsor, designated physician, or investigator will have access to the subject identification

code and the subject's genetic data. In addition, relevant regulatory authorities may require access to relevant documents.

# 12. STUDY MANAGEMENT

## 12.1 Data Handling and Records Retention

The investigator is responsible for retaining and managing all study documents in accordance with GCP requirements, including but not limited to the protocol, eCRF, and signed ICF. These documents should be kept on file at the site until 5 years after the end of the study.

Study documents should be properly preserved for future access or data traceability. Safety and environmental risks should be considered in records retention.

## 12.2 Raw Data/File Access

The investigator agrees that the sponsor, CRO, and relevant authorized regulatory authorities have direct access to all study-related documents, including subjects' medical records.

## 12.3 Changes to the Protocol

Any change to the protocol during the study must be approved by the sponsor and investigators before implementation.

All amendments of the protocol shall be maintained as supplements. Any change to the protocol shall be submitted to the Ethics Committee for approval, and amendments shall be approved or filing in accordance with ethics Committee regulations.

## 12.4 Responsibilities of Investigator

The investigator will conduct the study in strict accordance with the protocol, ICH, GCP, and local laws and regulations. The relevant detailed responsibilities of the investigators are listed in Chapter 5 (Responsibilities of the Investigators) of the Chinese GCP (Office Order No. 3).

## 12.5 Publication of Results

All data generated in this study are confidential. The sponsor has the right to publish the research results. Information on publication policies between each sponsor and investigator will be described in the clinical trial agreement

All information about this study (not limited to the following documents: study protocol, investigator's brochure) must be strictly confidential. The investigator must recognize that the scientific or medical information derived from the study may be of commercial value to the sponsor. Therefore, the information and data relevant to this study should be kept confidential by the investigator. Manuscripts, abstracts, or other modes of presentation arising from the results of the study must be reviewed and approved in writing by the sponsor in advance. In order to protecting the sponsor's proprietary, the

sponsor may request that the investigator refrain from publishing information about the study until the product is approved for marketing.

The sponsor has the right to publish information or data related to the study, or to report it to the drug administration. The sponsor must obtain the consent of the investigator for the use of the investigator's name in publication or advertising.

# 13 REFERENCES


[1] Wakai T, Shirai Y, Moroda T, Yokoyama N and Hatakeyama K. Impact of ductal resection margin status on long-term survival in patients undergoing resection for extrahepatic cholangiocarcinoma. CANCER-AM CANCER SOC. 2005; 103:1210-1216.

[2] Raderer M, Hejna MH, Valencak JB, Kornek GV, Weinlander GS, Bareck E, Lenauer J, Brodowicz T, Lang F, Scheithauer W. Two consecutive phase II studies of 5-fluorouracil/leucovorin/mitomycin C and of gemcitabine in patients with advanced biliary cancer. Oncology. 1999; 56:177–180.

[3] Choi CW, Choi IK, Seo JH, Kim BS, Kim JS, Kim CD, Um SH, Kim YH. Effects of 5-fluorouracil and leucovorin in the treatment of pancreatic-biliary tract adenocarcinomas. Am J Clin Oncol. 2000; 23:425–428.

[4] Ducreux M, Van Cutsem E, Van Laethem JL, Gress TM, Jeziorski K, Rougier P, Wagener T, Anak O, Baron B, Nordlinger B. A randomised phase II trial of weekly high-dose 5-fluorouracil with and without folinic acid and cisplatin in patients with advanced biliary tract carcinoma: results of the 40955 EORTC trial. Eur J Cancer. 2005; 41:398–403.

[5] Patt YZ, Hassan MM, Lozano RD, Hoque AM, Raijman I, Waugh KA. Phase II trial of cisplatin (P), Intron A (I), Adriamycin (A), and 5-fluorouracil (F)(PIAF) for biliary tree cancer (BTC) Proc Am Soc Clin Oncol 199918297a(abstract)

[6] Kajanti M, Pyrhonen S. Epirubicin-sequential methotrexate-5-fluorouracil-leucovorin treatment in advanced cancer of the extrahepatic biliary system. A phase II study. Am J Clin Oncol. 1994; 17:223–226.

[7] Valle JW, Wasan H, Johnson P, et al. Gemcitabine alone or in combination with cisplatin in patients with advanced or metastatic cholangiocarcinomas or other biliary tract tumours: a multicentre randomised phase II study — the UK ABC-01 Study. Br J Cancer 2009; 101:621-7.

[8] Valle J, Wasan H, Palmer DH, et al.Cisplatin plus gemcitabine versus gemcitabine for biliary tract cancer.N Engl J Med. 2010;362(14):1273－81.

[9] Randomized Phase III study of gemcitabine plus S-1 versus gemcitabine plus cisplatin in advanced biliary tract cancer: Japan Clinical Oncology Group Study (JCOG1113, FUGA-BT). ASCO GI.2018.

[10] Sahai V, Catalano PJ, Zalupski MM, et al. Nab-Paclitaxel and Gemcitabine as First-line Treatment of Advanced or Metastatic CholangiocarcinomaA Phase 2 Clinical Trial. JAMA Oncol. 2018, 1; 4(12): 1707-1712.

[11] Shroff RT, Javle MM, Xiao L, et al. Gemcitabine, Cisplatin, and nab-Paclitaxel for the Treatment of Advanced Biliary Tract Cancers: A Phase 2 Clinical Trial. JAMA Oncol. 2019, 1; 5(6): 824-830.

# Appendix 1

## Response Evaluation Criteria in Solid Tumors RECIST Version 1.1 (Excerpt)

### Measurability of tumour at baseline

Definitions

At baseline, tumour lesions/lymph nodes will be categorized measurable or non-measurable as follows:

3.1.1. Measurable

Tumour lesions: Must be accurately measured in at least one dimension (longest diameter in the plane of measurement is to be recorded) with a minimum size of:

• 10mm by CT scan (CT scan slice thickness no greater than 5 mm; see Appendix II on imaging guidance).
• 10mm caliper measurement by clinical exam (lesions which cannot be accurately measured with calipers should be recorded as non-measurable).
• 20mm by chest X-ray.

Malignant lymph nodes: To be considered pathologically enlarged and measurable, a lymph node must be P15mm in short axis when assessed by CT scan (CT scan slice thickness recommended to be no greater than 5 mm). At baseline and in follow-up, only the short axis will be measured and followed (see Schwartz et al. in this Special Issue[15]). See also notes below on 'Baseline documentation of target and non-target lesions' for information on lymph node measurement.

3.1.2. Non-measurable

All other lesions, including small lesions (longest diameter <10mm or pathological lymph nodes with P10 to <15mm short axis) as well as truly non-measurable lesions. Lesions considered truly non-measurable include: leptomeningeal disease, ascites, pleural or pericardial effusion, inflammatory breast disease, lymphangitic involvement of skin or lung, abdominal masses/abdominal organomegaly identified by physical exam that is not measurable by reproducible imaging techniques.

3.1.3. Special considerations regarding lesion measurability

Bone lesions, cystic lesions, and lesions previously treated
with local therapy require particular comment:

Bone lesions:

• Bone scan, PET scan or plain films are not considered adequate imaging techniques to measure bone lesions. However, these techniques can be used to confirm the presence or disappearance of bone lesions.
• Lytic bone lesions or mixed lytic-blastic lesions, with identifiable soft tissue components, that can be evaluated by cross sectional imaging techniques such as CT or MRI can be considered as measurable lesions if the soft tissue component meets the definition of measurability described above.
• Blastic bone lesions are non-measurable.

Cystic lesions:

• Lesions that meet the criteria for radiographically defined simple cysts should not be considered as malignant lesions (neither measurable nor non-measurable) since they are, by definition, simple cysts.
• 'Cystic lesions' thought to represent cystic metastases can be considered as measurable lesions, if they meet the definition of measurability described above. However, if noncystic lesions are present in the same patient, these are preferred for selection as target lesions.

Lesions with prior local treatment:

• Tumour lesions situated in a previously irradiated area, or in an area subjected to other loco-regional therapy, are usually not considered measurable unless there has been demonstrated progression in the lesion. Study protocols should detail the conditions under which such lesions would be considered measurable.

3.2. Specifications by methods of measurements

3.2.1. Measurement of lesions

All measurements should be recorded in metric notation, using calipers if clinically assessed. All baseline evaluations should be performed as close as possible to the treatment start and never more than 4 weeks before the beginning of the treatment.

3.2.2. Method of assessment

The same method of assessment and the same technique should be used to characterise each identified and reported lesion at baseline and during follow-up. Imaging based evaluation should always be done rather than clinical examination unless the lesion(s) being followed cannot be imaged but are assessable by clinical exam.

Clinical lesions: Clinical lesions will only be considered measurable when they are superficial and P10mm diameter as assessed using calipers (e.g. skin nodules). For the case of skin lesions, documentation by colour photography including a ruler to estimate the size of the lesion is suggested. As noted above, when lesions can be evaluated by both clinical exam and imaging, imaging evaluation should be undertaken since it is more objective and may also be reviewed at the end of the study.

Chest X-ray: Chest CT is preferred over chest X-ray, particularly when progression is an important endpoint, since CT is more sensitive than X-ray, particularly in identifying new lesions. However, lesions on chest X-ray may be considered measurable if they are clearly defined and surrounded by aerated lung. See Appendix II for more details.

CT, MRI: CT is the best currently available and reproducible method to measure lesions selected for response assessment. This guideline has defined measurability of lesions on CT scan based on the assumption that CT slice thickness is 5mm or less. As is described in Appendix II, when CT scans have slice thickness greater than 5 mm, the minimum size for a measurable lesion should be twice the slice thickness. MRI is also acceptable in certain situations (e.g. for body scans). More details concerning the use of both CT and MRI for assessment of objective tumour response evaluation are provided in Appendix II.

Ultrasound: Ultrasound is not useful in assessment of lesion size and should not be used as a method of measurement. Ultrasound examinations cannot be reproduced in their entirety for independent review at a later date and, because they are operator dependent, it cannot be guaranteed that the same technique and measurements will be taken from one assessment to the next (described in greater detail in Appendix II). If new lesions are identified by ultrasound in the course of the study, confirmation by CT or MRI is advised. If there is concern about radiation exposure at CT, MRI may be used instead of CT in selected instances.

Endoscopy, laparoscopy: The utilisation of these techniques for objective tumour evaluation is not advised. However, they can be useful to confirm complete pathological response when biopsies are obtained or to determine relapse in trials where recurrence following complete response or surgical resection is an endpoint.

# 4. Tumour response evaluation

## 4.3.1. Evaluation of target lesions

Complete Response (CR): Disappearance of all target lesions. Any pathological lymph nodes (whether target or non-target) must have reduction in short axis to <10 mm.

Partial Response (PR): At least a 30% decrease in the sum of diameters of target lesions, taking as reference the baseline sum diameters.

Progressive Disease (PD): At least a 20% increase in the sum of diameters of target lesions, taking as reference the smallest sum on study (this includes the baseline sum if that is the smallest on study). In addition to the relative increase of 20%, the sum must also demonstrate an absolute increase of at least 5 mm. (Note: the appearance of one or more new lesions is also considered progression).

Stable Disease (SD): Neither sufficient shrinkage to qualify for PR nor sufficient increase to qualify for PD, taking as reference the smallest sum diameters while on study.

## 4.3.2. Special notes on the assessment of target lesions

Lymph nodes. Lymph nodes identified as target lesions should always have the actual short axis measurement recorded (measured in the same anatomical plane as the baseline examination), even if the nodes regress to below 10mm on study. This means that when lymph nodes are included as target lesions, the 'sum' of lesions may not be zero even if complete response criteria are met, since a normal lymph node is defined as having a short axis of <10mm. Case report forms or other data collection methods may therefore be designed to have target nodal lesions recorded in a separate section where, in order to qualify for CR, each node must achieve a short axis <10mm. For PR, SD and PD, the actual short axis measurement of the nodes is to be included in the sum of target lesions. Target lesions that become 'too small to measure'. While on study, all lesions (nodal and non-nodal) recorded at baseline should have their actual measurements recorded at each subsequent evaluation, even when very small (e.g. 2mm). However, sometimes lesions or lymph nodes which are recorded as target lesions at baseline become so faint on CT scan that the radiologist may not feel comfortable assigning an exact measure and may report them as being 'too small to measure'. When this occurs it is important that a value be recorded on the case report form. If it is the opinion of the radiologist that the lesion has likely disappeared, the measurement should be recorded as 0mm. If the lesion is believed to be present and is faintly seen but too small to measure, a default value of 5mm should be assigned (Note: It is less likely that this rule will be used for lymph nodes since they usually have a definable size when normal and are frequently surrounded by fat such as in the retroperitoneum; however, if a lymph node is believed to be present and is faintly seen but too small to measure, a default value of 5mmshould be assigned in this circumstance as well). This default value is derived from the 5mm CT slice thickness (but should not be changed with varying CT slice thickness). The measurement of these lesions is potentially non-reproducible, therefore providing this default value will prevent false responses or progressions based upon measurement error. To reiterate, however, if the radiologist is able to provide an actual measure, that should be recorded, even if it is below 5mm. Lesions that split or coalesce on treatment. As noted in Appendix II, when non-nodal lesions 'fragment', the longest diameters of the fragmented portions should be added together to calculate the target lesion sum. Similarly, as lesions coalesce, a plane between them may be maintained that would aid in obtaining maximal diameter measurements of each individual lesion. If the lesions have truly coalesced such that they are no longer separable, the vector of the longest diameter in this instance should be the maximal longest diameter for the 'coalesced lesion'.

## 4.3.3. Evaluation of non-target lesions

This section provides the definitions of the criteria used to determine the tumour response for the group of non-target lesions.

While some non-target lesions may actually be measurable, need not be measured and instead should be assessed only qualitatively at the time points specified in the protocol. Complete Response (CR): Disappearance of all non-target lesions and normal is ation of tumour marker level. All lymph nodes must be non-pathological in size (<10mm short axis). Non-CR/Non-PD: Persistence of one or more non-target lesion(s) and/or maintenance of tumour marker level above the normal limits.

Progressive Disease (PD): Unequivocal progression (see comments below) of existing non-target lesions. (Note: the appearance of one or more new lesions is also considered progression).

## 4.3.4. Special notes on assessment of progression of non-target disease

The concept of progression of non-target disease requires additional explanation as follows:

When the patient also has measurable disease. In this setting, to achieve 'unequivocal progression' on the basis of the non-target disease, there must be an overall level of substantial worsening in non-target disease such that, even in presence of SD or PR in target disease, the overall tumour burden has increased sufficiently to merit discontinuation of therapy (see examples in Appendix II and further details below). A modest 'increase' in the size of one or more non-target lesions is usually not sufficient to quality for unequivocal progression status. The designation of overall progression solely on the basis of change in non-target disease in the face of SD or PR of target disease will therefore be extremely rare.

When the patient has only non-measurable disease. This circumstance arises in some phase III trials when it is not a criterion of study entry to have measurable disease. The same general concepts apply here as noted above, however, in this instance there is no measurable disease assessment to factor into the interpretation of an increase in non-measurable disease burden. Because worsening in non-target disease cannot be easily quantified (by definition: if all lesions are truly non-measurable) a useful test that can be applied when assessing patients for unequivocal progression is to consider if the increase in overall disease burden based on the change in non-measurable disease is comparable in magnitude to the increase that would be required to declare PD for measurable disease: i.e.an increase in tumour burden representing an additional 73% increase in 'volume' (which is equivalent to a 20% increase diameter in a measurable lesion). Examples include an increase in a pleural effusion from 'trace' to 'large', an increase in lymphangitic disease from localised to widespread, or may be described in protocols as 'sufficient to require a change in therapy'. Some illustrative examples are shown in Figs. 5 and 6 in Appendix II. If 'unequivocal progression' is seen, the patient should be considered to have had overall PD at that point. While it would be ideal to have objective criteria to apply to non-measurable disease, the very nature of that disease makes it impossible to do so, therefore the increase must be substantial.

## 4.3.5. New lesions

The appearance of new malignant lesions denotes disease progression; therefore, some comments on detection of new lesions are important. There are no specific criteria for the identification of new radiographic lesions; however, the finding of a new lesion should be unequivocal: i.e. not attributable to differences in scanning technique, change in imaging modality or findings thought to represent something other than tumour (for example, some 'new' bone lesions may be simply healing or flare of pre-existing lesions). This is particularly important when the patient's baseline lesions show partial or complete response. For example, necrosis of a liver lesion may be reported on a CT scan report as a 'new' cystic lesion, which it is not.

A lesion identified on a follow-up study in an anatomical location that was not scanned at baseline is considered a new lesion and will indicate

disease progression. An example of this is the patient who has visceral disease at baseline and while on study has a CT or MRI brain ordered which reveals metastases. The patient's brain metastases are considered to be evidence of PD even if he/she did not have brain imaging at baseline.

If a new lesion is equivocal, for example because of its small size, continued therapy and follow-up evaluation will clarify if it represents truly new disease. If repeat scans confirm there is definitely a new lesion, then progression should be declared using the date of the initial scan.

While FDG-PET response assessments need additional study, it is sometimes reasonable to incorporate the use of FDG-PET scanning to complement CT scanning in assessment of progression (particularly possible 'new' disease). New lesions on the basis of FDG-PET imaging can be identified according to the following algorithm:

a. Negative FDG-PET at baseline, with a positive FDG-PET at follow-up is a sign of PD based on a new lesion.

b. No FDG-PET at baseline and a positive FDG-PET at follow-up:

If the positive FDG-PET at follow-up corresponds to a new site of disease confirmed by CT, this is PD.

If the positive FDG-PET at follow-up is not confirmed as a new site of disease on CT, additional follow-up CT scans are needed to determine if there is truly progression occurring at that site (if so, the date of PD will be the date of the initial abnormal FDG-PET scan).

If the positive FDG-PET at follow-up corresponds to a pre-existing site of disease on CT that is not progressing on the basis of the anatomic images, this is not PD.

### 4.4.2. Missing assessments and inevaluable designation

When no imaging/measurement is done at all at a particular time point, the patient is not evaluable (NE) at that time point. If only a subset of lesion measurements are made at an assessment, usually the case is also considered NE at that time point, unless a convincing argument can be made that the contribution of the individual missing lesion(s) would not change the assigned time point response. This would be most likely to happen in the case of PD. For example, if a patient had a baseline sum of 50mm with three measured lesions and at follow-up only two lesions were assessed, but those gave a sum of 80 mm, the patient will have achieved PD status, regardless of the contribution of the missing lesion.

### 4.4.4. Special notes on response assessment

When nodal disease is included in the sum of target lesions and the nodes decrease to 'normal' size (<10 mm), they may still have a measurement reported on scans. This measurement should be recorded even though the nodes are normal in order not to overstate progression should it be based on increase in size of the nodes. As noted earlier, this means that patients with CR may not have a total sum of 'zero' on the case report form (CRF).

In trials where confirmation of response is required, repeated 'NE' time point assessments may complicate best response determination. The analysis plan for the trial must address how missing data/assessments will be addressed in determination of response and progression. For example, in most trials it is reasonable to consider a patient with time point responses of PR-NE-PR as a confirmed response.

Patients with a global deterioration of health status requiring discontinuation of treatment without objective evidence of disease progression at that time should be reported as 'symptomatic deterioration'. Every effort should be made to document objective progression even after discontinuation of treatment. Symptomatic deterioration is not a descriptor of an objective response: it is a reason for stopping study therapy. The objective response status of such patients is to be determined by evaluation of target and non-target disease as shown in Tables 1–3.

Conditions that define 'early progression, early death and inevaluability' are study specific and should be clearly described in each protocol (depending on treatment duration, treatment periodicity).

In some circumstances it may be difficult to distinguish residual disease from normal tissue. When the evaluation of complete response depends upon this determination, it is recommended that the residual lesion be investigated (fine needle aspirate/biopsy) before assigning a status of complete response. FDG-PET may be used to upgrade a response to a CR in a manner similar to a biopsy in cases where a residual radiographic abnormality is thought to represent fibrosis or scarring. The use of FDG-PET in this circumstance should be prospectively described in the protocol and supported by disease specific medical literature for the indication. However, it must be acknowledged that both approaches may lead to false positive CR due to limitations of FDG-PET and biopsy resolution/sensitivity.

For equivocal findings of progression (e.g. very small and uncertain new lesions; cystic changes or necrosis in existing lesions), treatment may continue until the next scheduled assessment. If at the next scheduled assessment, progression is confirmed, the date of progression should be the earlier date when progression was suspected.

**Table 1 – Time point response: patients with target (+/– non-target) disease.**

| Target lesions | Non-target lesions | New lesions | Overall response |
|---|---|---|---|
| CR | CR | No | CR |
| CR | Non-CR/non-PD | No | PR |
| CR | Not evaluated | No | PR |
| PR | Non-PD or not all evaluated | No | PR |
| SD | Non-PD or not all evaluated | No | SD |
| Not all evaluated | Non-PD | No | NE |
| PD | Any | Yes or No | PD |
| Any | PD | Yes or No | PD |
| Any | Any | Yes | PD |

CR = complete response, PR = partial response, SD = stable disease, PD = progressive disease, and NE = inevaluable.

**Table 2 – Time point response: patients with non-target disease only.**

| Non-target lesions | New lesions | Overall response |
|---|---|---|
| CR | No | CR |
| Non-CR/non-PD | No | Non-CR/non-PD[a] |
| Not all evaluated | No | NE |
| Unequivocal PD | Yes or No | PD |
| Any | Yes | PD |

CR = complete response, PD = progressive disease, and NE = inevaluable.

a 'Non-CR/non-PD' is preferred over 'stable disease' for non-target disease since SD is increasingly used as endpoint for assessment of efficacy in some trials so to assign this category when no lesions can be measured is not advised.

**Table 3 – Best overall response when confirmation of CR and PR required.**

| Overall response First time point | Overall response Subsequent time point | BEST overall response |
|---|---|---|
| CR | CR | CR |
| CR | PR | SD, PD or PR[a] |
| CR | SD | SD provided minimum criteria for SD duration met, otherwise, PD |
| CR | PD | SD provided minimum criteria for SD duration met, otherwise, PD |
| CR | NE | SD provided minimum criteria for SD duration met, otherwise NE |
| PR | CR | PR |
| PR | PR | PR |
| PR | SD | SD |
| PR | PD | SD provided minimum criteria for SD duration met, otherwise, PD |
| PR | NE | SD provided minimum criteria for SD duration met, otherwise NE |
| NE | NE | NE |

CR = complete response, PR = partial response, SD = stable disease, PD = progressive disease, and NE = inevaluable.

a If a CR is *truly* met at first time point, then any disease seen at a subsequent time point, even disease meeting PR criteria relative to baseline, makes the disease PD at that point (since disease must have reappeared after CR). Best response would depend on whether minimum duration for SD was met. However, sometimes 'CR' may be claimed when subsequent scans suggest small lesions were likely still present and in fact the patient had PR, not CR at the first time point. Under these circumstances, the original CR should be changed to PR and the best response is PR.

4.6. Confirmatory measurement/duration of response

4.6.1. Confirmation

In non-randomised trials where response is the primary endpoint, confirmation of PR and CR is required to ensure responses identified are not the result of measurement error. This will also permit appropriate interpretation of results in the context of historical data where response has traditionally required confirmation in such trials (see the paper by Bogaerts et al. in this Special Issue[10]). However, in all other circumstances i.e. in randomised trials (phase II or III) or studies where stable disease or progression are the primary endpoints, confirmation of response is not required since it will not add value to the interpretation of trial results. However, elimination of the requirement for response confirmation may increase the importance of central review to protect against bias, in particular in studies which are not blinded.

In the case of SD, measurements must have met the SD criteria at least once after study entry at a minimum interval (in general not less than 6–8 weeks) that is defined in the study protocol.

4.6.2. Duration of overall response

The duration of overall response is measured from the time measurement criteria are first met for CR/PR (whichever is first recorded) until the first date that recurrent or progressive disease is objectively documented (taking as reference for progressive disease the smallest measurements recorded on study). The duration of overall complete response is measured from the time measurement criteria are first met for CR until the first date that recurrent disease is objectively documented.

4.6.3. Duration of stable disease

Stable disease is measured from the start of the treatment (in randomised trials, from date of randomisation) until the criteria for progression are met, taking as reference the smallest sum on study (if the baseline sum is the smallest, this is the reference for calculation of PD).

The clinical relevance of the duration of stable disease varies in different studies and diseases. If the proportion of patients achieving stable disease for a minimum period of time is an endpoint of importance in a particular trial, the protocol should specify the minimal time interval required between two measurements for determination of stable disease.

Note: The duration of response and stable disease as well as the progression-free survival are influenced by the frequency of follow-up after baseline evaluation. It is not in the scope of this guideline to define a standard follow-up frequency. The frequency should take into account many parameters including disease types and stages, treatment periodicity and standard practice. However, these limitations of the precision of the measured endpoint should be taken into account if comparisons between trials are to be made.

**Appendix 2**

Eastern Cooperative Oncology Group Performance Status (ECOG-PS)

| Scale | Performance status |
|---|---|
| 0 | Fully active, able to carry on all predisease activities without restriction |
| 1 | Restricted in physically strenuous activity but ambulatory and able to carry out work of a light or sedentary nature (e.g., light housework, office work). |
| 2 | Ambulatory and capable of all self-care but unable to carry out any work activities. Up and about more than 50% of waking hours. |
| 3 | Capable of only limited self-care, confined to bed or chair 50% or more of waking hours. |
| 4 | Completely disabled. Cannot carry on any self-care. Totally confined to bed or chair. |
| 5 | Death. |

# Statistical Analysis Plan

## A Phase 2, Clinical research on PD-1 inhibitor plus Gemcitabine based chemotherapy as the treatment in advanced biliary tract cancer

| | |
|---|---|
| **Protocol Version and Date:** | Version 1.0 Sep. 5, 2019 |
| **Phase:** | Phase 2 |
| **Methodology:** | Single-Arm Study |
| **Sponsor:** | Eastern Hepatobiliary Surgery Hospital Second, Military Medical University |
| **Analysis Plan Date:** | |
| **Analysis Plan Version:** | Version 1.0 |

**TABLE OF CONTENTS**


1. INTRODUCTION ........ 30
2. STUDY OBJECTIVES ........ 30
3. STUDY DESIGN AND PLAN ........ 30
4. DETERMINATION OF SAMPLE SIZE ........ 31
5. STUDY ENDPOINTS ........ 32
5.1. Primary Endpoints ........ 32
5.2. Secondary Endpoints ........ 32
5.3. Exploratory Endpoints ........ 32
6. GENERAL ANALYSIS CONSIDERATIONS ........ 32
6.1. Data Reporting ........ 32
6.2. Data Analysis and Summaries ........ 32
6.3. Data Handling ........ 32
6.3.1. Baseline Characteristics ........ 32
6.3.2. Partial Dates ........ 33
6.3.3. Standard Calculations ........ 33
7. ANALYSIS POPULATIONS ........ 34
7.1. Intent-to-Treat (ITT) Population ........ 34
7.2. Full Analysis Set (FAS) ........ 34
7.3. Safety Set (SS) ........ 34
8. STUDY POPULATION ........ 34
8.1. Subject Disposition ........ 34
8.2. Protocol Deviations ........ 34
8.3. Demographic and Baseline Characteristics ........ 34
8.4. Prior and New Concomitant Medications ........ 35
9. EFFICACY ANALYSE ........ 36
9.1. Primary Efficacy Analyses ........ 36
9.2. Secondary Efficacy Analyses ........ 36
9.2.1. ORR ........ 36
9.2.2. PFS ........ 36
9.2.3. DCR ........ 36
9.2.4. DOR ........ 36
9.3. Exploratory Analyses ........ 37
10. SAFETY ANALYSES ........ 37
10.1. Drug Exposure ........ 37
10.2. Adverse Events (AEs) ........ 37

10.3. Clinical Laboratory Evaluations ... 38
10.4. Vital Signs, physical examinations, and other safety-related examinations ... 38
10.5. 12-lead Electrocardiograms (ECGs) ... 38
11. COMPLIANCE ANALYSIS ... 38
12. INTERIM ANALYSIS ... 38

# 1. INTRODUCTION

This document outlines the statistical methods to be implemented during the analyses of the study phase data collected within the scope of Protocol (Clinical research on PD-1 inhibitor plus Gemcitabine based chemotherapy as the treatment in advanced biliary tract cancer). The purpose of this plan is to provide specific guidelines from which the analyses will proceed. Any deviations from these guidelines will be documented in the clinical study report (CSR). The scope of this plan includes the detailed specifications of the statistical analyses for the study only. The analyses described in this plan are considered a priori, in that they have been defined prior to database lock of study. Post hoc analyses will be labeled as such on the outputs and identified in the CSR. Further details about study design and procedures can be found in the protocol.

# 2. STUDY OBJECTIVES

The primary objective is to evaluate the efficacy of sintilimab combined with Gemcitabine -based chemotherapy as first-line therapy in patients with advanced biliary tract cancer.

The secondary objectives are to evaluate the safety and feasibility of sintilimab combined with Gemcitabine -based chemotherapy as first-line therapy in patients with advanced biliary tract cancer.

# 3. STUDY DESIGN AND PLAN

This is a single-arm clinical study aimed to evaluate the clinical efficacy and safety of sintilimab combined with Gemcitabine-based chemotherapy as the first-line therapy in patients with advanced biliary tract cancer. The trial design is shown in Figure 1.

30 treatment-naive Patients who pathologically confirmed biliary tract cancer, provide written informed consent and meet all of the inclusion and none of the exclusion criteria will be enrolled in this study.

For the first 6-8 cycles after enrollment, the subject's dose will be 1000 mg/m² gemcitabine and 25 mg/m² cisplatin on days 1 and 8, respectively, per 21-day cycle, as well as 200 mg Sintilimab intravenously on day 1 of each 21-day cycle. This combination regimen was sustained until disease progression, intolerable toxicity, or completion of 6–8 cycles of the treatment. The maintenance treatment with 200 mg sintilimab will continue for up to 2 years.

Subjects will have a screening period of up to 30 days prior to Day 0. Subjects will accept examination every 3 weeks, including blood routine, coagulation function, liver and kidney functional  electrolytes, tumor indicators, immune-related indicators and so on.   Tumor imaging evaluations will be assessed every 6 weeks. AEs will be reported from the time subjects first dose until 30 days after the last dose.

**Figure 1: Study Design Procedures**

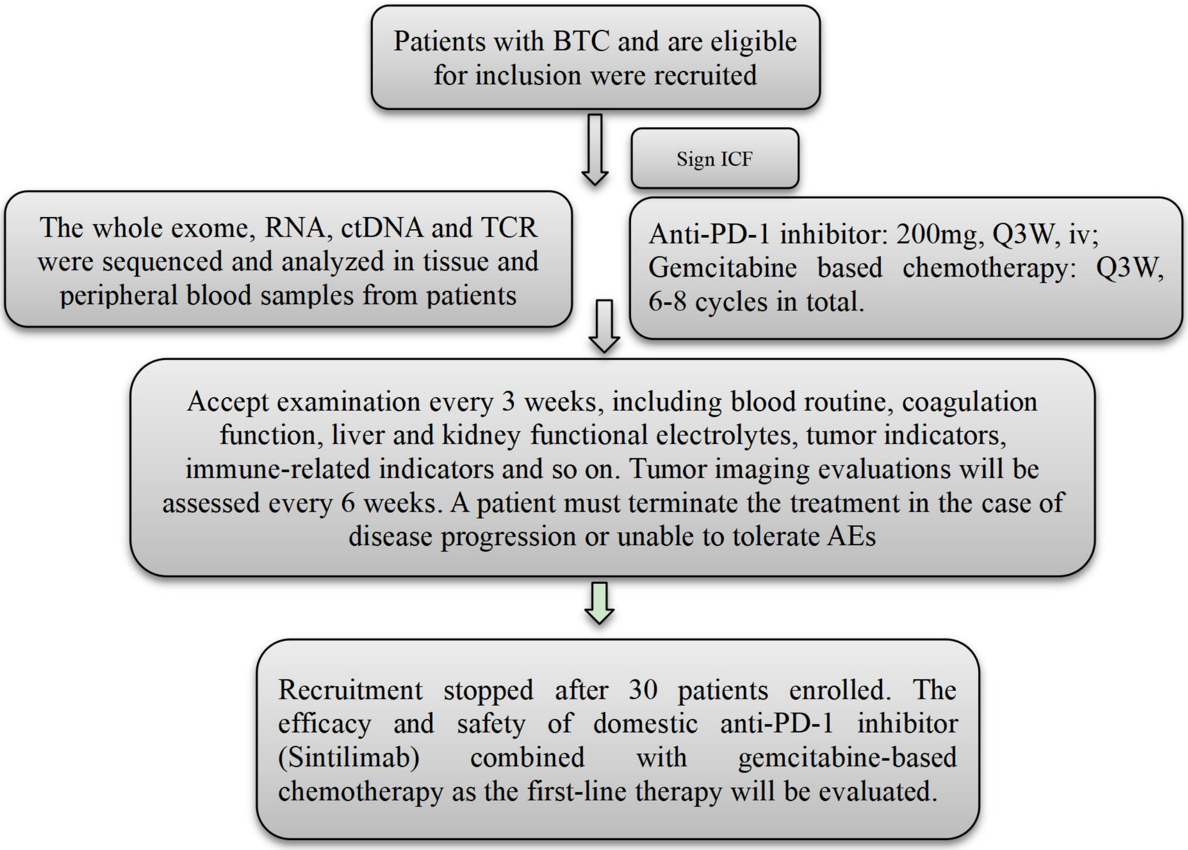


# 4. DETERMINATION OF SAMPLE SIZE

A sample size of 30 subjects is designed to evaluate efficacy and safety without statistical hypothesis.

# 5. STUDY ENDPOINTS

## 5.1. Primary Endpoints

The primary efficacy endpoint is Overall survival (OS).

## 5.2. Secondary Endpoints

As defined in the protocol the secondary endpoints are Objective response rate（ORR）， progression-free survival （PFS），Disease control rate（DCR），Duration of response (DOR) and safety.

## 5.3. Exploratory Endpoints

Correlation between biomarkers and therapeutic response to therapy.

# 6. GENERAL ANALYSIS CONSIDERATIONS

## 6.1. Data Reporting

The statistical analyses will be reported using summary tables, figures, and data listings.

Individual subject data obtained from the case report forms (CRFs), external laboratory data, and any derived data (such as change from Baseline and percent change from Baseline) will be presented in data listings by subject. Data from all assessments, whether scheduled or unscheduled, will be listed by subject and visit. Unscheduled visits and visits occurring more than one day outside protocol defined window will not be included in the summaries.

All statistical analyses were performed using SAS (V9.2 or higher).

## 6.2. Data Analysis and Summaries

Unless otherwise specified, variable data will be described using the mean ± standard deviation or median (maximum and minimum), and attributes data will be described using frequency and percentage. 95% confidence intervals were calculated if necessary.

## 6.3. Data Handling

### 6.3.1. Baseline Characteristics

For baseline characteristics, parameters are defined as the most recent no missing values prior to administration

of investigational product on Day 1. No missing value estimation.

### 6.3.2. Partial Dates

If only a partial date is available and is required for a calculation, the following standards will be applied:

- Date (If the date record is incomplete and does not affect logic)
  - For missing day only: Day will be imputed as the 15th day of the month if does not contradict another date.
  - For missing day and month: Day and month will be imputed as 01 July if does not contradict another date.
  - For missing day, month and year: no missing value estimation.
- Efficacy
  - All missing of primary efficacy Measurements due to withdrawal were included in the analysis as "not evaluable”.
  - When calculating the time variables (e.g., PFS, TTR, DOR), subjects with missing tumor assessment after treatment will be checked on a case-by-case basis to determine the deletion time during data audit.
- Safety
  - No missing value estimation.

### 6.3.3. Standard Calculations

Variables requiring calculation will be derived using the following formulas:

- **Days**: A duration expressed in days between one date (*date1*) and another later date (*date2*) will be calculated using the following formulas:

  duration (days) = date2 –date l + 1

- **Months**: A duration expressed in months is calculated as the number of days divided by 365.25 / 12.
- **Years**—A duration expressed in years between one date (*date1*) and another date (*date2*) is calculated using the following formulas:

  duration (years) = (date2 - date1+ 1) / 365.25

- **Age** — Age is calculated as the number of years from the date of birth (*DOB*) to the date of informed consent (*DOIC*). The following formula is used:

age (years) = year of DOIC - year of DOB +1.

# 7. ANALYSIS POPULATIONS

The analysis population includes intent to treat (ITT) analysis set, full analysis set (FAS), and safety set (SS).

## 7.1. Intent-to-Treat (ITT) Population

All subjects who receive any amount of investigational product will be included in the ITT population. The ITT population will be used for the analysis of all efficacy data.

## 7.2. Full Analysis Set (FAS)

Subset of the ITT analysis set, including the patients who had measurable lesions at baseline, and who have received at least one dose of the study drug. This dataset will be used as the primary analysis data set for the efficacy assessment of ORR.

## 7.3. Safety Set (SS)

The Safety population will include all subjects who receive any amount of investigational product. Treatment assignment will be based on the treatment actually received. The Safety population will be used for the analysis of all safety data.

# 8. STUDY POPULATION

## 8.1. Subject Disposition

Subject disposition information will be summarized and listed for all subjects. The number and percentage of subjects enrolled, completed or early terminated will be summarized.

## 8.2. Protocol Deviations

Protocol deviations for missed visits, missed assessments, out of window visits or assessments, and violations of inclusion/exclusion criteria will be determined based on available data. All other protocol deviations will be collected.

## 8.3. Demographic and Baseline Characteristics

Demographic variables will include the following:

- Age at informed consent
- Sex

Other Baseline characteristics will include the following:

- History of cancer

Disease term, cholangiocarcinoma location, pathological type, clinical stage, pathological stage, metastatic site, etc.,

- Baseline height and weight (BMI, body surface area)
- Baseline vital signs: systolic blood pressure, diastolic blood pressure, pulse, temperature, respiration, ECOG PS score
- Baseline target and non-target lesions: number of lesions, total diameter, and distribution of lesion sites
- Virus detection and hepatitis B 5 items, HBV DNA, HCV RNA

Demographic and Baseline characteristics will be summarized for the ITT populations. The patients' demographic characteristics (gender, age), tumor diagnosis information (pathological diagnosis, clinical staging), and other baseline information (height, and weight [body mass index]), vital signs, ECOG PS, and laboratory tests will be analyzed using descriptive statistics.

## 8.4. Prior and New Concomitant Medications

Verbatim terms on CRFs will be mapped to ATC class and preferred term using the World Health Organization Drug Dictionary Enhanced (WHO-DDE June 2014).

Pretreatment medications: medications with start and stop dates prior to the first dose of investigational product.

Prior concomitant medications: medications that started prior to, and continued after, the first dose of investigational product.

New concomitant medications: medications that were started after the first dose of investigational product.

If it cannot be determined whether the medication was a new concomitant medication due to a partial start or stop date or if the medication is taken on the same date as the first dose in, then it will be counted as a new concomitant medication.

Pretreatment medications will be presented in listings only. Prior and new concomitant medications will be summarized by World Health Organization ATC class and preferred term using the ITT Population. New concomitant medications will be summarized separately. These summaries will present the number and percentage of subjects using each medication. Prior and new concomitant medications will be presented a data

listing.

# 9. EFFICACY ANALYSE

The primary and secondary efficacy analyses will be based on the ITT Population.

## 9.1. Primary Efficacy Analyses

The primary efficacy endpoint is OS on the ITT population. Overall survival (OS): Time from first dose to death recorded for any cause. Patients who are still alive at the time of analysis are censored at the last contacted date. The inter-group comparison of OS will be performed using the stratified log-rank test, the median OS and corresponding 95% CI will be estimated using the Kaplan-Meier method, and the survival curves will be plotted.

## 9.2. Secondary Efficacy Analyses

Secondary efficacy are Objective response rate (ORR), progression-free survival （PFS）， disease control rate (DCR), and duration of response (DOR).

### 9.2.1. ORR

ORR assessed by the investigator: According to RECIST V1.1, the proportion of patients who achieve CR or PR assessed in the analysis population.

### 9.2.2. PFS

PFS assessed by the investigator: According to RECIST V1.1, the time from first dose to the first recorded imaging disease progression or death caused by any reason as assessed by the investigator, whichever occurs first. Patients who are still alive with no disease progression record at the time of analysis are censored at the last imaging evaluation date. Patients who are still alive with no imaging evaluation record after baseline are censored at the first dose date.

### 9.2.3. DCR

DCR assessed by the investigator: According to RECIST V1.1, the proportion of patients who achieve CR, PR, or SD assessed by the investigator in the analysis population.

### 9.2.4. DOR

DOR assessed by the investigator: According to RECIST V1.1, the time from the first recorded CR or PR to disease progression or death for patients with CR or PR assessed by the investigator. Patients who are still alive with no disease progression at the time of analysis are censored at the last imaging evaluation. The median DOR will be estimated via the Kaplan-Meier method and the survival plots will be plotted.

## 9.3. Exploratory Analyses

Correlation between biomarkers and therapeutic response to therapy. Multiomics biomarkers associated with clinical response were assessed as an exploratory objective.

# 10. SAFETY ANALYSES

All safety analyses will be based on the SS, with safety parameters in including AEs, laboratory tests, vital signs, 12-lead ECG, etc.

## 10.1. Drug Exposure

The drug exposure and duration of treatment (number of treatment cycles) will be summarized. The duration of investigational product exposure will be calculated as Follows:

- Exposure to investigational product (week= {[(Date of last investigational product dose-Date of 1st investigational product dose) + 1] - Total duration of temporary investigational product discontinuation}

## 10.2. Adverse Events (AEs)

All AEs will be coded and classified using MedDRA, and graded as per CTCAE V5.0.

All AE summaries will be restricted to treatment-emergent adverse events (TEAEs), which are defined as any AEs that newly appear, increase in frequency, or worsen in severity following initiation of study medication. The incidences (frequency) of all TEAEs, TEAEs at grade 3 and above, TEAEs related to the study drugs, irAEs, SAEs, SAEs related to study drugs, TEAEs leading to discontinued study medication, and TEAEs leading to study termination will be summarized, and the above-mentioned AEs will be summarized based on SOCs and PTs in MedDRA coding. In addition, the severity levels of TEAEs and relationship with the study drug were also summarized by SOC and PT.

The incidence of pre-treatment AEs and pre-treatment SAEs occurring after ICF signoff and before the first dosing of investigational product (OCA or placebo) will be tabulated in the same manner as above for all subjects participating in the washout period.

The following listings will be presented by subject:

- All AEs
- Serious AEs (subset of the AEs where serious is marked as “Yes”)
- Death information will be provided in a separate listing, should any deaths occur
- Severe AEs (subset of AEs where severity is marked as “Severe” or severity is missing)

- Related AEs (subset of AEs where relationship to study medication is marked as “Definite”, “Possible” or “Probable”)
- AE's leading to withdrawal of investigational product (subset of AEs where action taken with study medication is marked as “Drug Withdrawn”)
- AE's leading to Study Discontinuation (subset of AEs where subject discontinued from study is checked)

## 10.3. Clinical Laboratory Evaluations

A listing of available laboratory reference/normal ranges for each laboratory parameter will be provided including age, sex, values with units. For laboratory tests, the observed values and changes from the baseline will be analyzed using descriptive statistics. The baseline results and the worst results during the trial were presented in a crosstab. Laboratory test abnormalities will be graded and summarized according to CTCATE V5.0.

## 10.4. Vital Signs, physical examinations, and other safety-related examinations

Measured values and changes from baseline for vital signs, physical examinations, and other safety-related examination values will be analyzed using descriptive statistics. The baseline results and the worst results during the trial were presented in a crosstab.

ECOG and PS will be analyzed and summarized using descriptive statistics.

## 10.5. 12-lead Electrocardiograms (ECGs)

Descriptive statistics are used for 12-lead ECG and changes from baseline. A cross-classification table is used to describe ECG and changes from baseline before and after the treatment and data lists will be provided.

# 11. COMPLIANCE ANALYSIS

The proportion and frequency of patients violating the expected administration regimen, the proportion of patients in whom the doses of study drugs account for between 80–120% of the those prescribed by the protocol, the proportion of patients completing the study, and the proportion of patients completing different treatment cycles will be summarized.

# 12. INTERIM ANALYSIS

The interim analysis will not be carried out in this study
