# Supplementary material for: Clinical and biomarker analyses of sintilimab plus gemcitabine and cisplatin as first-line treatment for patients with advanced biliary tract cancer
Source: Nat Commun. 2023 Mar 11;14:1340. doi: 10.1038/s41467-023-37030-w (PMC10008621; doi:10.1038/s41467-023-37030-w)
Supplement: Supplementary file 2 — Description of Additional Supplementary Files [file 41467_2023_37030_MOESM2_ESM.pdf]

Description of Additional Supplementary Files

Supplementary Data 1: Nanostring normalized gene expression data.
