# Supplementary material for: Clinical and biomarker analyses of sintilimab plus gemcitabine and cisplatin as first-line treatment for patients with advanced biliary tract cancer
Source: Nat Commun. 2023 Mar 11;14:1340. doi: 10.1038/s41467-023-37030-w (PMC10008621; doi:10.1038/s41467-023-37030-w)
Supplement: Supplementary file 4 — Reporting Summary [file 41467_2023_37030_MOESM4_ESM.pdf]

nature portfolio

Corresponding author(s): Zhen-gang Yuan

Last updated by author(s): Feb 22, 2023

Reporting Summary

Nature Portfolio wishes to improve the reproducibility of the work that we publish. This form provides structure for consistency and transparency in reporting. For further information on Nature Portfolio policies, see our Editorial Policies and the Editorial Policy Checklist.

Statistics

For all statistical analyses, confirm that the following items are present in the figure legend, table legend, main text, or Methods section.

n/a	Confirmed	
☐	☒	The exact sample size (n) for each experimental group/condition, given as a discrete number and unit of measurement
☐	☒	A statement on whether measurements were taken from distinct samples or whether the same sample was measured repeatedly
☐	☒	The statistical test(s) used AND whether they are one- or two-sided<br>*Only common tests should be described solely by name; describe more complex techniques in the Methods section.*
☐	☒	A description of all covariates tested
☐	☒	A description of any assumptions or corrections, such as tests of normality and adjustment for multiple comparisons
☐	☒	A full description of the statistical parameters including central tendency (e.g. means) or other basic estimates (e.g. regression coefficient) AND variation (e.g. standard deviation) or associated estimates of uncertainty (e.g. confidence intervals)
☐	☒	For null hypothesis testing, the test statistic (e.g. F, t, r) with confidence intervals, effect sizes, degrees of freedom and P value noted<br>*Give P values as exact values whenever suitable.*
☒	☐	For Bayesian analysis, information on the choice of priors and Markov chain Monte Carlo settings
☒	☐	For hierarchical and complex designs, identification of the appropriate level for tests and full reporting of outcomes
☐	☒	Estimates of effect sizes (e.g. Cohen's d, Pearson's r), indicating how they were calculated

Our web collection on statistics for biologists contains articles on many of the points above.

Software and code

Policy information about availability of computer code

Data collection	Statistical analyses and graph illustration were performed using SAS 9.4 (SAS Institute, Cary, NC, USA) and R 4.1.3.
Data analysis	Survival was analyzed using Kaplan–Meier curves and log-rank test, and values of p < 0.05 were considered to be statistically significant in the remaining statistical analyses. We calculated 95% CI using the exact Clopper–Pearson method.

For manuscripts utilizing custom algorithms or software that are central to the research but not yet described in published literature, software must be made available to editors and reviewers. We strongly encourage code deposition in a community repository (e.g. GitHub). See the Nature Portfolio guidelines for submitting code & software for further information.

Data

Policy information about availability of data

All manuscripts must include a data availability statement. This statement should provide the following information, where applicable:

- Accession codes, unique identifiers, or web links for publicly available datasets
- A description of any restrictions on data availability
- For clinical datasets or third party data, please ensure that the statement adheres to our policy

The raw genomic sequencing data generated in this study have been deposited in the Genome Sequence Archive in National Genomics Data Center, China National Center for Bioinformation under accession code HRA003603 (https://bigd.big.ac.cn/gsa-human/browse/HRA003603). The raw sequencing data are available under restricted access due to data privacy laws, upon request for 1 years. Data are available on request sharing by sending requests to the corresponding author Zhen-gang Yuan (yuanzg@smmu.edu.cn), which will need the approval of the institutional ethical committees. Access can be obtained by completing the application form

March 2021

via GSA-Human System. Clinical data were not publicly available due to involving patient privacy, but can be accessed from the corresponding author, upon request for 3 years; individual de-identified patient data will be shared for clinical study analyses. The remaining data are available in the manuscript, Supplementary Information, or Source Data file. The study protocol is provided in the Supplementary Information file. Source data are provided with this paper.

Human research participants

Policy information about studies involving human research participants and Sex and Gender in Research.

Reporting on sex and gender	18 (60%) of the 30 patients were male in this study.
Population characteristics	The baseline demographics of the enrolled patients are presented in sTable 4. The median age was 56.5 years (range: 35–73). Patients with an Eastern Cooperative Oncology Group performance score of 0–1 accounted for 96.66% (29/30) of patients; 18 (60%) of the 30 patients were male, while 25 (83.3%) had metastatic disease. Intrahepatic cholangiocarcinoma was mostly found to be of the primary tumor type (28/30, 93.3%).
Recruitment	30 treatment-naive Patients with advanced biliary tract cancer will be enrolled in this study.<br>Recreitment Criteria:<br>1. Histologically, cytologically confirmed biliary tract cancer;<br>2. with at least one measurable lesion as the target lesion according to the Response Evaluation Criteria in Solid Tumors (RECIST) criteria (V.1.1);<br>3. Patients aged 18 to 75;<br>4. An Eastern Cooperative Oncology Group performance status of 0–2;<br>5. Adequate haematological function (white blood cell count > 3.0 x10^9/L, platelet count > 100 x10^9/L);<br>6. Adequate liver function (bilirubin ≤ 1.5 times the upper limit of normal (ULN);<br>7. Adequate renal function (creatinine clearance > 60 mL /min; creatinine < 120μmol /L);<br>8. Had no heart failure, no uncontrollable chest pain, and no myocardial infarction within 12 months prior to the study;<br>9. Expected survival≥3 months;<br>10. Signed an informed consent form.
Ethics oversight	This was a single-armed, open-labeled, phase II, prospective study (https://www.chictr.org.cn/showproj.aspx?proj=59003), preregistered on 24th Aug, 2020. The study was conducted according to the guidelines dictated by the Helsinki Declaration and international standards of good clinical practice. The Ethics Committee of Eastern Hepatobiliary Surgery Hospital approved the protocol and any protocol amendments.

Note that full information on the approval of the study protocol must also be provided in the manuscript.

Field-specific reporting

Please select the one below that is the best fit for your research. If you are not sure, read the appropriate sections before making your selection.

☒ Life sciences ☐ Behavioural & social sciences ☐ Ecological, evolutionary & environmental sciences

For a reference copy of the document with all sections, see nature.com/documents/nr-reporting-summary-flat.pdf

Life sciences study design

All studies must disclose on these points even when the disclosure is negative.

Sample size	This was a single-arm study, and no randomization was used. To determine the sample size for this clinical trial, overall survival (OS) with standard of care chemotherapy (Gem/Cis) as the historical control was assumed to be 9.5 months based on previously reported data of Asian population56 and the notable features of the high proportion of ICC patients in our center. The addition of sintilimab to chemotherapy would expect to improve the OS to 16.0 months. Given an accrual period of 24 months, a maximum follow-up time of 48 months, at the significance level of 0.05, to achieve the power of 0.8, the number of events required is 23. Equivalently, a sample size of 30 is needed57. The sample size result is based on a one-sided test with exponential assumption for survival time.
Data exclusions	Data exclusions:<br>1. Received systemic treatment previously, including chemotherapy, targeted and immunotherapy; 2. Associated secondary malignancies or other type of tumors with metastasis to the brain or meninges within 3 years prior to study initiation;<br>3. Drug contraindications: severe hypertension, high risk of bleeding, severe nephrotic syndrome, etc.;<br>4. With chronic diarrhea or colorectal inflammatory conditions, or untreated obstruction or incomplete obstruction affecting systemic administration;<br>5. Active infection or other severe infection that may prevent the patient from receiving planned management (bacterial cholangitis, which destroys a branch of the biliary tract over a long period of time without control);<br>6. Cardiac insufficiency, unstable angina pectoris, congestive heart failure, myocardial infarction occurred within 6 months before enrollment, serious uncontrollable arrhythmia; 6. Participated in other clinical trials;<br>7. Have a history of uncontrollable substance abuse or mental disorder;<br>8. Patients with concomitant diseases that, in the investigator's judgment, may seriously endanger their own safety or may interfere with the completion of the study;

	9. Participated in other clinical trials;<br>10. Pregnant or lactating women;<br>11.Individuals under corrective monitoring or supervision.
Replication	IHC staining were performed one time in 25 independent samples with similar results. Multiple immunofluorescence staining were performed one time in 29 independent samples with similar results, responders (n=11) and non-responders (n=18)
Randomization	Phase 2 single arm, non-randomization trial.
Blinding	The investigators were blinded to group allocation during data collection and/or analysis.

Reporting for specific materials, systems and methods

We require information from authors about some types of materials, experimental systems and methods used in many studies. Here, indicate whether each material, system or method listed is relevant to your study. If you are not sure if a list item applies to your research, read the appropriate section before selecting a response.

Materials & experimental systems

n/a	Involved in the study	
☐	☒	Antibodies
☒	☐	Eukaryotic cell lines
☒	☐	Palaeontology and archaeology
☒	☐	Animals and other organisms
☐	☒	Clinical data
☒	☐	Dual use research of concern

Methods

n/a	Involved in the study	
☒	☐	ChIP-seq
☒	☐	Flow cytometry
☒	☐	MRI-based neuroimaging

Antibodies

Antibodies used	The following primary antibodies were used for immunostaining: anti-CD4 (Ventana, SP35, 1:100), anti-CD8 (Ventana, SP57, 1:400), and anti-PD-L1 (Ventana, SP263, 1:250) antibodies. We used 4',6-diamidino-2-phenylindole (Sigma) to stain the nuclei.
Validation	Multiple immunofluorescence staining were performed one time in 29 independent samples with similar results, responders (n=11) and non-responders (n=18).

Clinical data

Policy information about clinical studies

All manuscripts should comply with the ICMJEguidelines for publication of clinical research and a completedCONSORT checklist must be included with all submissions.

Clinical trial registration	ChiCTR2000036652
Study protocol	uploaded already
Data collection	This was a single-armed, open-labeled, phase II, prospective study (https://www.chictr.org.cn/showproj.aspx?proj=59003), preregistered on 24th Aug, 2020. The study was conducted according to the guidelines dictated by the Helsinki Declaration and international standards of good clinical practice. The Ethics Committee of Eastern Hepatobiliary Surgery Hospital approved the protocol and any protocol amendments. All the enrolled patients provided a written informed consent form. In this study, we enrolled 30 patients with advanced BTC receiving GemCis plus sintilimab between August 2020 and May 2022 in the Shanghai Eastern Hepatobiliary Surgery Hospital. Newly diagnosed 18–75-year-old patients with histologically and cytologically confirmed BTC were eligible for inclusion. Furthermore, patients with at least one measurable lesion as the target lesion according to RECIST V.1.1 and an Eastern Cooperative Oncology Group performance status of 0–2 were eligible for inclusion. The exclusion criteria for the patients were as follows: the presence of secondary malignancies or other types of tumors with metastasis to the brain or meninges within 3 years before study initiation and incidence of concomitant diseases that, in the investigator's judgment, may seriously endanger their safety or interfere with the completion of the study.
Outcomes	The primary endpoint was OS, which indicates the day from the first dose of GemCis or sintilimab to death from any cause. Secondary endpoints included progression-free survival (PFS; the day from the first dose to disease progression or death), ORR, the proportion of complete response rate plus partial response (PR) rate under computerized tomography), and disease control rate [DCR; the proportion of patients who achieved CR plus PR and stable disease (SD)]. As to render safety, adverse events (AEs) in the entire study process were reported as per CTCAE V.5.0. Imaging and pathology were confirmed by two clinical specialists. Investigators evaluated the responses based on RECIST V.1.1. Toxicity profile included the events occurring 30 days after the end of therapy in all patients. Multiomics biomarkers associated with clinical response were assessed as an exploratory objective.
